# What a pain in the back: etiology, diagnosis and future treatment directions for discogenic low back pain

**DOI:** 10.1038/s41413-025-00472-7

**Published:** 2025-10-21

**Authors:** Giselle Kaneda, Lea Zila, Jacob T. Wechsler, Karim Shafi, Karandeep Cheema, Hyun Bae, Sang D. Kim, Alexander Tuchman, Debiao Li, Dmitriy Sheyn

**Affiliations:** 1 Orthopaedic Stem Cell Research Laboratory, Cedars-Sinai Medical Center, Los Angeles, CA USA; 2 Board of Governors Regenerative Medicine Institute, Cedars-Sinai Medical Center, Los Angeles, CA USA; 3 Department of Biomedical Sciences, Cedars-Sinai Medical Center, Cedars-Sinai Medical Center, Los Angeles, CA USA; 4 Keck School of Medicine, University of Southern California, Los Angeles, CA USA; 5 Department of Orthopaedics, Cedars-Sinai Medical Center, Los Angeles, CA USA; 6 Biomedical Imaging Research Institute, Cedars-Sinai Medical Center, Los Angeles, CA USA; 7 Department of Bioengineering, University of California, Los Angeles, CA USA; 8 Department of Surgery, Cedars-Sinai Medical Center, Los Angeles, CA USA

**Keywords:** Pathogenesis, Diseases

## Abstract

Chronic lower back pain (LBP) is the leading cause of disability worldwide. Due to its close relationship with intervertebral disc (IVD) degeneration (IVDD), research has historically focused more on understanding the mechanism behind IVDD while clinical efforts prioritize pain management. More recently, there has been a shift toward understanding LBP as a distinct pathological entity. This review synthesizes current knowledge on discogenic LBP, combining known pathophysiology, molecular mechanisms, risk factors, diagnostic challenges, and available experimental models. IVDD is a complex, multifactorial process involving biochemical, mechanical, and inflammatory changes within the disc, leading to structural breakdown and potential discogenic pain. Key mechanisms include extracellular matrix degradation, upregulation of inflammatory mediators, immune cell infiltration, and aberrant nerve and vascular ingrowth. However, not all cases of IVDD result in LBP, highlighting the need for further investigation into the cellular, molecular, and biomechanical factors contributing to symptom development. Current diagnostic tools and experimental models for studying discogenic LBP remain limited, impeding the development of targeted treatments. Existing therapies primarily focus on symptom management rather than addressing underlying disease mechanisms.

## Introduction

Chronic lower back pain (LBP) is the greatest cause of disability burden worldwide, affecting an estimated 619 million people.^1^ This figure is projected to increase to 843 million by 2050, largely due to population aging and expansion.^1^ Of patients reporting chronic LBP, approximately 40% are attributed to intervertebral disc (IVD) degeneration, and as a result, IVD degeneration (IVDD) has traditionally been the focus of research rather than LBP itself.^2^ However, in clinical practice, it is LBP that often takes precedence over IVDD. Consequently, there has been a shift towards exploring LBP as an entity distinct from its link to IVDD. This transition underscores the importance of delving into the intricacies of LBP as a multifaceted condition with its own unique mechanisms and clinical implications. In our recent study, we tackled the question of the cellular and molecular differences between degenerated asymptomatic and LBP-inducing IVDs.^3^ In this review we will take a wider look at this problem and elaborate on what is known to date and what open questions remain to be answered.

### IVD anatomy, degeneration process, and discogenic low back pain

#### IVD unique structure and anatomy

The IVD is an organ situated between two vertebral bodies, comprising the nucleus pulposus (NP) at its center, the annulus fibrosus (AF) encircling the NP, and a thin layer of fibrocartilage that separates the NP from the vertebral body endplates. Each component serves distinct functions essential for spinal function and biomechanics.

The NP, primarily responsible for shock absorption and flexibility, is composed mainly of water absorbing extracellular matrix (ECM) and a small population of cells. The ECM within the NP is predominantly made up of collagens and proteoglycans, with collagen II and aggrecan being the primary constituents.^4^ Collagen II functions to form irregular networks of fibers in the IVD matrix to provide disc hydration, structure, and elasticity.^5^ Aggrecan is credited with the ability to retain water and thus is thought to be largely responsible for preserving water content, osmotic pressure, and biomechanical properties within the NP.^4^ Although the cells within the NP constitute only about 1% of its total volume, they play a crucial role in maintaining disc homeostasis.^6^ These cells are responsible for synthesizing ECM proteins, cytokines, growth factors, and proteases that contribute to the NP’s integrity and functionality.

Surrounding the NP is a ring of highly organized connective tissue known as the AF, which provides structural stability to the disc. In humans, the AF is organized into 15 to 25 stacked layers known as lamellae, which are composed primarily of collagen I.^7^ Within each lamella, collagen fibers are aligned in parallel with an orientation that shifts by about 60° between each adjacent layer to enhance the disc’s resistance to compressive forces.^7^ Between the lamellae are AF cells, which are responsible for producing ECM, including proteoglycans, elastic fibers, and glycoproteins.

A transition zone, known as the inner AF, sees a gradual shift in ECM properties and cell composition that bridges the gap from the organized outer AF to the less rigid NP. Cells within the transition zone exhibit a rounder morphology, in contrast to the spindle-shaped cells characteristic of the AF. Additionally, the ECM composition exhibits a reduction in the ratio of collagen I to collagen II as one approaches the NP. This gradual transition facilitates a smooth gradation in mechanical properties between the rigid outer AF and the flexible NP.^8^

At the rostro-caudal boundaries of the IVD are endplates composed of a bone-cartilage bilayer connecting the disc to adjacent vertebrae. These endplates are vital for maintaining disc homeostasis, with their damage or calcification linked to IVDD.^9^ Given the IVD’s largely avascular nature, exchange of nutrients and waste predominantly takes place through the endplate and the outer edge of the AF.^10^

#### Intervertebral disc degeneration process, causes, and symptoms

The relationship between LBP and IVDD has long been known, but the exact mechanism linking these phenomena has yet to be identified. IVDD is a multifactorial disease, and progression is challenging to stop once degenerative changes begin. Among various contributing factors, age is thought to be the most influential factor on IVDD. Signs of IVDD have been observed in pre-pubescent children and exist in about 20% of teenagers, with the percentage steadily increasing with age.^11^ As the disc ages, the homeostatic balance between anabolic and catabolic processes within the ECM gradually becomes disrupted, resulting in a net reduction of ECM content and quality within the IVD.^12,13^ This imbalance can be further exacerbated by injury to the disc, whether from acute, chronic, or even micro damage.^14^

During IVDD, changes in the cellular phenotype of IVD cells, especially in the NP, are observed.^15^ These changes are triggered by various forms of cell stress, including mechanical, metabolic, and oxidative stress.^16,17^ IVD-resident cells, namely NP cells (NPC) and AF cells (AFC), which are phenotypically unique, balance the breakdown and production of the disc’s matrix under healthy conditions.^5^ However, during IVDD, the metabolic balance within the disc is disrupted, resulting in a shift toward net catabolism.^18^ At the molecular level, early indicators of IVDD manifest through the decreased production and accelerated breakdown of the disc’s ECM, including critical components like proteoglycans and collagen II.^19^ Upregulation of Matrix Metalloproteases (MMPs) and ADAMTS (A Disintegrin and Metalloproteinase with Thrombospondin Motifs) family are a likely culprit for this change in ECM composition. MMPs, ADAMTSs, and other proteases designed to degrade ECM are typically expressed at low levels to maintain normal tissue turnover.^20,21^ However, as IVDD develops, proteases including MMP3 and MMP13, ADAMTS1 and ADAMTS4 become upregulated thus increasing the rate of ECM degradation.^20,21^

Though the IVD is considered an immune-privileged site, recent single-cell RNA sequencing (scRNA-seq) studies have identified small subsets of native immune cells within the disc.^22–24^ In our own study, human cells injected to a healthy rat IVD did not induce immune response or rejection.^25^ In the early stages of degeneration, immune cells were found to adopt anti-inflammatory and tissue repair roles, whereas in later stages, macrophages tend to exhibit a more proinflammatory phenotype.^24^ Specifically, fragmented ECM components are recognized by resident immune cells as damage-associated molecular patterns (DAMPs), triggering additional immune cell infiltration into the IVD. These immune cells, including macrophages and neutrophils, release cytokines such as TNF-α and IL-1, further amplifying the inflammatory response within the disc. Whether immune cells enter the disc freely or through pre-existing fissures, has yet to be determined.

Inflammation within the IVD is a key contributor to the development and progression of IVDD.^26^ While inflammation can have both positive and negative effects on disease progression, in the context of IVDD, it is seen as detrimental.^26^ Changes in the IVD microenvironment induce cell stress, prompting the expression of pro-inflammatory factors, including cytokines, chemokines, and neurotrophins.^27^ Interestingly, many of these factors have a dual role in not only initiating an inflammatory response but also in recruiting immune cells, thereby perpetuating the inflammatory cascade within the disc.^27^ Indeed, expression of inflammatory markers such as TNFs and interleukins have been found to be significantly upregulated in degenerated discs and are believed to exacerbate the degenerative process by further attracting inflammatory cells into the disc microenvironment.^27^ The influx of pro-inflammatory cytokines into the IVD induces changes in collagen makeup and loss of proteoglycans.^17^ IL-1β and TNF-α downregulate the production of collagen II and aggrecan. As IVDD progresses, collagen II is replaced by collagen I, which is more organized and decreases the effectiveness of nutrient diffusion to NPCs. A decrease in aggrecan levels leads to dehydration and increased stiffness of the disc, altering the biomechanical properties of the NP.^17^ As the NP becomes dehydrated, its capacity to absorb shock and evenly distribute pressure across the disc diminishes. Consequently, the AF is subjected to greater compressive and shear forces, escalating the risk of disc damage. Damage during this phase often extends to the endplates, impairing nutrient supply and waste removal. This impairment can trigger cellular stress, potentially leading to cell senescence, damage, or even death, along with a reduction in the pH of the disc.^28^ We have shown that annular injury and consequent IVDD can also lead to changes in pH in a porcine model.^29–31^ Research indicates that a lower pH environment within the disc significantly contributes to tissue inflammation and cellular stress, leading to an increase in MMP3, Interleukin (IL)-6, IL-8, Tumor Necrosis Factor (TNF) Alpha, Nerve Growth Factor (NGF), and Connective Tissue Growth Factor (CTGF) secretion.^3^ This acidic environment may also exacerbate ECM degradation by altering matrix metabolism, affecting cell activity, and even inducing cell death.^28^

### IVD degeneration induces discogenic low back pain

Due to the multifaceted nature of LBP, it can be difficult to pinpoint the exact source of pain. In fact, a majority of LBP cases do not have a clear pathoanatomical cause, which makes developing treatments and therapies especially challenging.^32–34^ Besides the IVD, many other structures have been associated with LBP development, including the vertebral endplate, spasm or strain of spinal muscles, spondylosis, and other spinal conditions such as spinal stenosis and disc herniation.^10,32,35–38^

**Fig. 1:**
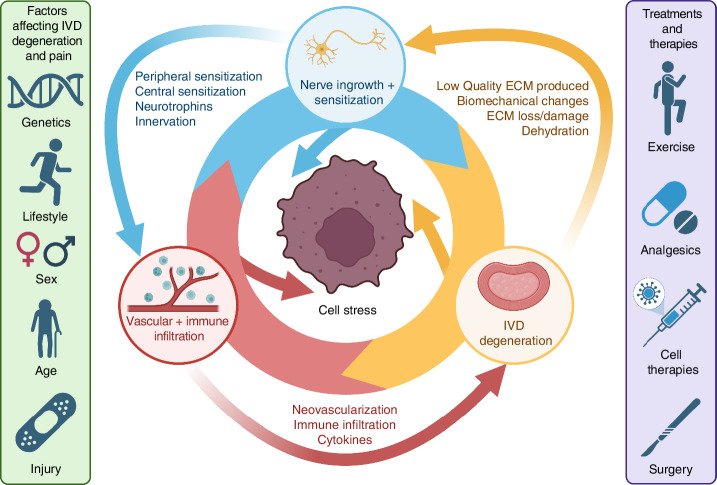
Proposed relationship between IVD degeneration, angiogenesis and immune cell infiltration, and nerve ingrowth and sensitization

The development of LBP has long been considered synonymous with IVDD, with up to 40% of LBP cases attributed to this condition.^39,40^ However, studies have found that depending on a patient’s age, between 30% and 95% of IVDD cases are asymptomatic.^41^ Discogenic LBP is a multifactorial disease, with various factors implicated in its development, including hyperinnervation, vascularization, and inflammation of the IVD (Fig. 1).^8^

In healthy discs only the outer AF is innervated, leaving the inner AF and NP aneural.^42^ For a degenerated disc to exhibit discogenic pain, innervation is required, but the exact locations of this innervation and the factors driving pain development are not well understood. Studies have shown an increased innervation extent and depth within degenerated discs is associated with LBP, suggesting that hyperinnervation may be a key factor.^42^ In our study, we have demonstrated the link between nociceptors and discogenic pain induction in in vitro and in vivo models.^3^

Healthy IVDs are also predominantly avascular in adults. During childhood, the disc is moderately vascularized; however, by age 17, this vasculature dies off.^11^ Despite this, it has been noted that as IVDD progresses, neovascularization of the outer layers of the AF occurs.^43^

Unfortunately, despite its high prevalence and disability burden, the exact mechanism governing the development of IVDD has yet to be elucidated. Our lack of understanding of both degeneration and discogenic LBP has limited our ability to develop effective preventative treatments, and thus, current treatments for LBP focus on treating a patient’s symptoms rather than the disease mechanism.

### The goal of the current review and its structure

Our current understanding and treatments for LBP are limited. A significant amount of work has been done to elucidate the biology and pathology of IVDD. However, the question of how and why LBP develops in some cases has yet to be determined.

IVDD and LBP are chronic conditions that are widely considered a natural part of aging.^11,44,45^ However, the chronicity of IVDD makes it more challenging to study relative to acute diseases, necessitating the use of long-term studies that require increased funds, supplies, and staff. In addition, chronic diseases often include more variables for consideration, such as genetics, sex, environmental, and lifestyle factors.

Current treatments for LBP are limited, primarily aiming to manage symptoms through analgesics or, in severe cases, surgical intervention, which treat the symptoms but not the mechanism of disease. This approach is largely due to a limited understanding of the specific processes by which discogenic LBP occurs, highlighting a critical need for advancements in our understanding and treatment of this condition.

Several reviews have examined different aspects of LBP; however, a comprehensive synthesis of key elements relevant to research and treatment approaches remains lacking. Some reviews provide a broad overview of LBP but do not discuss the models and methods used to study the condition.^46^ Others focus on LBP while omitting discussion of the molecular mechanisms that may contribute to its development.^47^ Additionally, some reviews address the mechanisms underlying LBP in humans, but do not include any animal models that can provide increased insight into exact molecular mechanisms or assess current treatment strategies that can be used to address them.^48^

To address this gap in literature, we have identified key advancements, tools and methodologies through targeted searches to synthesize a comprehensive narrative review of discogenic LBP and its relationship with IVDD. This review will discuss the current clinical definition of discogenic LBP and its relationship with IVDD, emphasizing our understanding of molecular mechanisms and risk factors that may contribute to its development. It will also outline available methodologies, experimental models, and current treatments. Further, this review aims to integrate our current understanding of cellular and molecular component in IVDD induction^3^ and methodologies to create a comprehensive resource for researchers in the field by providing a structured overview of existing literature while identifying challenges and areas for further investigation. The integration of biological, clinical, and translational perspectives presented in this review may facilitate the development of more effective, targeted treatments for LBP.

This review specifically focuses on discogenic LBP, and the methods currently employed to study it. However, it must be acknowledged that other non-discogenic pathologies can and often do contribute to LBP.

Specifically, this review seeks to define the current methods for diagnosing discogenic LBP and the challenges faced by research and clinical communities. Additionally, it explores the etiology of discogenic LBP, examining established and potential mechanisms underlying its development. It further discusses the models and evaluation methods used to study LBP and IVDD. Finally, the review explores current treatment strategies and potential future therapeutic approaches.

## Defining and diagnosing discogenic pain

Despite the high prevalence of LBP in the population today, the definition of “discogenic” pain alone is vague and varies even amongst clinicians. Often referred to as “axial” back pain, this type of pain is typically viewed as a combination of tears in the AF, introduction of nociceptive nerve endings, and cytokine irritation causing an inflammatory cascade, all exacerbated by mechanical loading. These factors create an environment that creates a variable pain presentation, consisting of stiffness, midline LBP, and radicular/neuropathic symptoms. As such, no universally accepted definition of discogenic LBP exists. However, clinicians often use a combination of consistent symptoms, physical examination findings, imaging studies, and other diagnostic modalities to identify the pathologic process in an individual with LBP.

Perhaps the most widely accepted clinical presentation of discogenic LBP is an active individual in their 2^nd^ through 5^th^ decade of life who presents predominantly axial LBP with or without intermittent radicular symptoms.^49–51^ These symptoms are classically exacerbated by activities that load the anterior column, including sitting or standing, and they typically experience relief when supine. Motion or physical activity that loads the disc in a standing or flexed position, including weightlifting activities, running, or sports, may be particularly painful. Identification of any “red flag” findings must be evaluated since occult trauma, infection, or malignancy may present in similar, nonspecific ways.^52^ Physical examination is typically unremarkable, as discogenic LBP is unlikely to cause neurologic deficits in the absence of compression (e.g., herniated disc). Exacerbation of pain with forward flexion is classically thought to be driven by the disc, whereas painful lumbar extension is thought to be driven by the facet joints and posterior elements, so-called “facetogenic pain.” However, the specificity for painful lumbar flexion and hyperextension is rather poor, and this finding alone is insufficient to diagnose discogenic pain accurately.^52^

Confirmation of discogenic LBP may be done through either non-invasive or invasive modalities. Non-invasive modalities include history taking, physical examination, and imaging studies. X-ray remains the first line imaging study in evaluating persistent LBP and provides an overall view of global alignment and lumbar pathology.^53^ Findings such as transitional anatomy, spondylolisthesis or spondylolysis, and loss of disc height may be quickly identified on a lateral lumbar X-ray and provide the clinician with important clues to the etiology of the patient’s pain.^52^ However, there remains a poor correlation between imaging findings and reported symptoms, as upwards of 30% of patients with axial LBP have normal X-rays. As such, the present indication for plain film X-rays for LBP includes mechanical symptoms without any “red flag” findings that persist for greater than 1 month.

Judicious use of Magnetic Resonance Imaging (MRI) is required to help tease out the diagnosis of discogenic LBP, given that degenerative disc changes and varying degrees of spinal stenosis are found in asymptomatic individuals.^50^ T1- and T2-weighted images on MRI provide excellent visualization of the IVD, neural elements, and surrounding soft tissues, but this abundance of detail has led to drastic overuse in modern medicine, particularly amongst non-spine specialists.^54^ Many MRI findings within the IVD, including height loss, annular fissures, loss of high signal intensity within the disc on T2-weight imaging, anterolisthesis/retrolisthesis, and bulging of the NP, are not associated with pain.^55–57^ Some degree of disc degeneration has been reported in 44%–90% of individuals from the 2nd to 7th decades of life.^55,56,58,59^ Assessment of the disc may be reported as a Pfirrmann score, which grades the disc based on the signal intensity within the NP on axial T2-weighted images and on disc height on sagittal images.^60^ However, this grading is typically used in a research setting, as clinicians may simply refer to a degenerated disc as a so-called “black disc”.^61^ Nevertheless, MRI findings must be carefully assessed in the setting of presenting symptoms in order to determine their clinical significance. Annular tears may be identified by a High-Intensity Zone (HIZ) of the posterior AF. HIZ are assessed on a sagittal T2 weighted MRI of the lumbar spine and are defined by an area of high signal intensity (white) along the posterior annulus with a low signal intensity (black) border that has been shown to have a correlation with LBP and findings of provocative discography.^62^

Recently, new MRI techniques have been introduced in an attempt to clarify the degenerative process within the IVD through quantitative methods. Quantitative MRI utilizes T2 relaxation time as a quantitative assessment for intervertebral disc degeneration. By evaluating subtle changes in disc structure and composition, this study shows early promise as a means of evaluating regional changes in the AF.^53,63,64^ T1-Rho is another MRI technique introduced to detect early disc degeneration based on loss of proteoglycans and shows some promise in its ability to detect early disc degeneration.^65,66^ Similarly, delayed gadolinium-enhanced MRI of cartilage (dGemeric) is an MRI study protocol that provides quantitative analysis of cartilage and has been applied to the assessment of IVDs.^67^ Endplate cartilage integrity and disc glycosaminoglycan (GAG) concentration can also theoretically be assessed via sodium MRI.^68^ Quantitative chemical exchange saturation transfer (qCEST) MRI is another emerging modality capable of detecting pH changes in IVDs and has been indirectly correlated with pain.^29–31^ While these novel quantitative metrics may offer clinicians valuable insight into the early degenerative process, further research is needed to provide a full understanding of these imaging modalities before their clinical relevance may be determined.

Perhaps the most controversial invasive diagnostic modality for discogenic back pain is provocative discography. This procedure has been foundational in research and the clinical evaluation of discogenic pain.^69^ Discography is performed by injection of iodine-based contrast into the subject disc in question, and the dye pattern, volume of injection, and patient’s pain response are recorded. However, despite its long history of use, its validity has never been clearly established. Perhaps the most significant limitations of this study are the reliability of the patient’s response and operator variability. Multiple studies have attempted to establish the positive predictive value of provocative discography and have been plagued by an unacceptably high false positive rate given the multimodal nature of LBP from a psychosomatic, social, and neurologic standpoint.^61,69–71^ In addition, the safety of discography remains a significant concern, as iatrogenic disc degeneration has been postulated to be a potential consequence of provocative discography. Other significant risks include discitis, disc herniation, nerve damage, or allergic reactions to the contrast medium.^61,72^ A 2014 guideline update for the performance of fusion procedure for degenerative disease of the lumbar spine provided a Grade C recommendation for the use of discography in patient selection. This consensus noted that while discography may be considered a diagnostic option in the management of LBP, it should not be used as a stand-alone diagnostic tool.^73^ Recent advances in imaging techniques such as qCEST offer promising, less invasive alternatives for evaluating discogenic pain.^29–31^ Taken in sum, clinicians must carefully weigh the risks and benefits of discography as a diagnostic tool and ensure careful selection of patients with whom they feel may benefit from this study.

## Etiology of discogenic pain

Historically, research into IVDD and LBP has centered around environmental lifestyle factors that induced histological changes within the disc. More recently, however, with the rise of genetic testing, research has begun to explore other factors posing a potential risk of IVDD and LBP development such as genetic factors, sex, high BMI, smoking, and ergonomic factors. However, the exact relationship between these factors on discogenic LBP has yet to be fully elucidated. As a result, understanding discogenic LBP etiology and why it only occurs in a subset of IVDD patients is not understood.

### Genetics, environmental, and lifestyle risk factors for discogenic pain

#### Biological sex as a risk factor

IVDD and LBP have widely been considered diseases stemming from environmental and lifestyle factors and thus was studied without consideration of how sex may impact the development, presentation, and progression of the disease. Recent studies into how sex differences affect these conditions have unveiled complex interactions involving hormonal fluctuations, anatomical distinctions, and life stages such as pregnancy and menopause that may play a role in LBP development.^74,75^ Sex has long been shown to impact LBP development with females having a higher global prevalence than males across all age groups and differences becoming more pronounced at older ages.^1,75^

MRI studies examining sex differences in IVDD and LBP revealed that females have lower NP-T1ρ values and more severe disc degeneration than males at any given age.^76,77^ The NP-T1ρ value is a marker of proteoglycan content and disc health, suggesting that, on average, males may maintain healthier disc composition into older age.^76,77^

A landmark study by Mosley et al. highlighted stark sex-based differences in the experience and biological markers of IVDD and LBP in a rat model.^78,79^ While minor differences were noted in the onset and progression of IVDD between sexes, the study revealed profound disparities in pain perception and the expression of pain-related markers.^78,79^ Male rats exhibited a significant reduction in pain sensitivity (as measured by paw withdrawal) starting at 2 weeks post-injury and persisting to sacrifice at 6 weeks post-injury.^79^ In comparison, females not only showed no significant changes in pain sensitivity post-injury but also had significantly more variability both at baseline and after injury, which could not be attributed solely to baseline differences or the estrus cycle.^79^ Furthermore, only males showed significant upregulation of pain markers in DRGs.^79^ These findings suggest that sex plays a critical and complex role in the manifestation of LBP, underpinned by biological and possibly genetic differences.

This sex-specific variability is particularly important to consider, given that many studies investigating LBP using animal models often overlook sex as a biological variable, opting to only use one sex or disregard sex in mixed-sex cohorts. However, the distinct responses observed between male and female animals emphasize the necessity of considering sex as a crucial factor in pain research, prompting a more nuanced exploration of the underlying mechanisms driving sex-related differences in LBP manifestation.

#### Genetic predisposition

While a diverse array of studies has explored the role of genetics in IVDD, there remains a notable gap in understanding how genetic predisposition may influence the development of LBP. Early studies involving mono- and dizygotic twins have highlighted a strong association between genetic background and the development of IVDD.^80–82^ More recently, the advent of genetic testing and genome-wide association studies (GWAS) have emerged as promising tools to identify genes and polymorphisms associated with LBP. A range of genes, including SLC13A1 and FSCN3, as well as specific polymorphisms such as rs12310519 in SOX5 and rs2228570 and rs7975232 in VDR, have been implicated in LBP.^83–86^

Baumbauer et al. conducted a study integrating genomic and pain testing, identifying the rs4680 genotype (GG) of catecholamine-O-methyltransferase (COMT), an enzyme involved in catecholamine metabolism, as being associated with an increased risk of transitioning from acute to chronic back pain.^87^ Additionally, patients with the rs4680 genotype exhibited heightened sensitivity to cold and mechanical stimuli.^87^

Beyond single nucleotide polymorphisms (SNPs) located in protein-coding regions of the genome, SNPs located in non-exon coding regions have also been implicated in LBP. Variants such as intergenic variant rs7833174, situated between CCDC26 and GSDMC, and intronic variant rs4384683, found within the DCC gene, have been associated with LBP.^84^

Furthermore, while numerous gene polymorphisms have been linked to IVDD, including those affecting ECM proteins, cytokines, and ECM degradation enzymes, their roles in contributing to LBP remain uncertain.^88^ For instance, polymorphisms within the IL-1 gene have demonstrated associations with both increased and decreased susceptibility to LBP.^89,90^

The genetic landscape influencing LBP is complex, encompassing both exonic and non-exonic variants, and involves multiple biological pathways. Despite advancements in genetic research, the precise mechanisms by which genetic predisposition contributes to LBP are not fully elucidated. Continued research integrating genomic data with clinical phenotypes is crucial to uncover the nuanced genetic factors at play and develop personalized treatment strategies for those affected by LBP.

#### Lifestyle factors

Lifestyle factors are some of the most important factors, outside of age, that can predict IVDD development. Factors such as physical activity, body weight, age, posture, occupational hazards, nutrition, stress, sleeping habits, and smoking have all been linked to an increased risk of LBP.^75^

Back pain is a significant issue for athletes and former athletes, particularly those involved in specific sports. LBP is especially prevalent among gymnasts. A meta-analysis by Trompeter et al. revealed that both male and female gymnasts have a high lifetime prevalence of LBP, exceeding 65%, with male gymnasts experiencing an even higher rate of over 80%.^91^ Other sports with a high risk of long-term LBP (affecting over 60% of participants) include rowing, weightlifting, and wrestling.^91^ The excessive spinal loading and high training volumes associated with these sports are key risk factors for overuse injuries, leading to chronic pain and degeneration.

Gymnastics sees a high prevalence of LBP among athletes under the age of 18. The sport demands extreme spinal movements such as flexion, torsion, and hyperextension, combined with high-impact activities.^92^ A study on adolescent female gymnasts found that 45% experienced LBP, with higher rates among those who had reached menarche. Post-menarche skeletal development, including increases in weight and height, contributes to additional spinal stress.

Excessive axial loading of the spine is another major factor in chronic LBP. Sports like powerlifting, weightlifting, and CrossFit, which involve heavy lifting, commonly lead to spinal injuries such as IVD bulges, herniations, and muscle strains.^93^ Weightlifters have a notably high lifetime incidence of LBP, exceeding 20%.

Repetitive lumbar flexion also contributes to LBP in athletes. In rowers, those with LBP exhibit distinct kinematic patterns compared to their pain-free counterparts. Rowers with LBP show increased lumbar spine range of motion (ROM) and decreased hip ROM during the catch phase of a stroke, leading to greater posterior pelvic tilt and reduced engagement of spinal extensor muscles.^94^ Conversely, healthy rowers maintain a more neutral pelvic tilt, greater hip ROM during the catch phase, and better co-activation of trunk and spine extensor muscles, resulting in improved spine stabilization during the pull.^94^ The increased load exerted onto the spine when the erector muscles are not properly engaged can be attributed to the flexion relaxation phenomenon. This biomechanical phenomenon occurs because of spinal flexion and is characterized by a sudden reduction in the myoelectrical activity of the paraspinal muscles, supporting the spine.^95^ Due to lack of engagement of the trunk extensors, the bending load is transferred onto passive tissues, including ligaments and IVDs, making the individual more susceptible to disc injury.^95^

While overexertion and improper spine loading can lead to LBP, sedentary behaviors are also linked to chronic LBP. Sedentary lifestyles, characterized by increased sitting time and decreased physical activity, are associated with higher obesity rates.^96^ Prolonged sitting has been shown to be a significant risk factor for LBP development in adults, likely due to postural changes in the spine over time, resulting in muscle atrophy and disc degeneration. Additionally, sedentary behaviors closely correlate with obesity, which further influences LBP development. Excess body weight increases the load on spinal tissues, potentially causing overload and damage. Obesity can also exacerbate LBP through other mechanisms, such as increases in systemic inflammation. Hypertrophied adipose tissue is characterized by chronic upregulation of pro-inflammatory cytokines like TNF, IL-6, and IL-1B, leading to long-term low-grade inflammation.^97^ Engaging in any form of exercise (while avoiding over-exercise) is proposed as a potential conservative treatment for patients with LBP who led sedentary lifestyles. Exercise can alleviate symptoms by reducing body fat, lowering inflammatory markers, and increasing trunk muscle strength.^97^

Chronic LBP is a significant cause of disability and impaired quality of life, particularly prevalent in older adults. Among individuals over 60 years of age, the prevalence of LBP ranges from 40% to 70%. The severity of LBP increases with age, with those over 80 experiencing pain that is three times more severe than that of younger individuals.^98^ In the aging population, LBP has no single pathology and often arises from multiple sources, including disc degeneration, facet joint pain, lumbar degenerative spondylosis, and other issues in the areas surrounding the lumbar spine.^98^

### The role of cytokines, inflammation, and vascularization in discogenic LBP

One of many factors contributing to the onset and propagation of IVDD is the activation of inflammatory pathways through pro-inflammatory molecules known as cytokines. The pathways can be activated through both intracellular and extracellular mechanisms.

Pathogen-associated molecular patterns (PAMPs) and damage-associated molecular patterns (DAMPs) are critical activators of the inflammatory response in IVDD. PAMPs, which arise from microbial infections, viruses, and fungi, are less often associated with IVDD propagation.^99–101^ DAMPs are more commonly responsible for triggering the inflammatory cascades associated with IVDD and include, but are not limited to, cellular stress or injury, ECM degradation products, mitochondrial dysfunction, and mechanical stress.^102^ DAMPs are molecules that are released by stressed or damaged cells and can originate from intracellular components such as mitochondrial DNA, ATP, uric acid crystals, and HMGB1.^103–106^ They can also be released from extracellular matrix fragments, including hyaluronan fragments and fibronectin.^107–109^ Additional ECM fragments have been reported in the literature as DAMPs. Fragments from collagen II have been found in multiple studies to induce cytokine and MMP production.^110–115^ Also, aggrecan has been reported to induce chemokine CCL2 release by recruiting immune cells to the disc.^116^ Other less common proteoglycans, including fragments from biglycan, decorin, and versican, have also been identified as DAMPs.^117–123^ Additionally, cell stress and death via mechanical overloading, environmental stress, cellular aging, and senescence release intracellular DAMPs, further adding to inflammatory signaling.

DAMPs are recognized as danger signals by specialized receptors known as pattern recognition receptors (PRRs).^103,124^ These receptors can be expressed by immune cells such as macrophages and dendritic cells, as well as non-immune cells, including those that make up the NP and AF in the disc.^99,125–127^ The DAMP molecules contribute to the upregulation of cytokines such as IL-1, IL-6, IL-8, IL-17, TNF-α, and interferon-γ (IFN-γ) and have long been linked to IVDD.^27,128–134^ DAMPs initiate the activation of intracellular multi-protein complexes known as inflammasomes by binding to PRRs, such as the toll-like receptor (TLR).^135^ Upon DAMP binding, the TLR recruits intracellular adaptor proteins, primarily Myeloid Differentiation Primary Response Protein 88 (MyD88).^136^ MyD88 acts as a bridge, connecting the activated receptor complex to downstream signaling molecules. It undergoes a change in shape and recruits IL-1 receptor-associated kinase 4 (IRAK4), which then phosphorylates and activates IRAK1. Activated IRAK1 dissociates from the receptor complex and interacts with downstream signaling molecules, leading to the activation of the Transforming Growth Factor-beta-Activated Kinase 1 (TAK1) complex.^135^ TAK1, a serine/threonine kinase, activates multiple downstream signaling pathways, including nuclear factor kappa-light-chain-enhancer activated B cells (NF-κB) and mitogen-activated protein kinase (MAPK) pathways.^99,137^ Downstream, NF-κB translocates to the nucleus, inducing the transcription of pro-IL-1β and inactive NLRP3, a component of the inflammasome, making it a potential therapeutic target.^138^ The NLRP3 protein is then produced and released from the nucleus, along with the inactive pro-IL-1β.^139^ Once the inactive NLRP3 protein is released, a secondary signal induces a conformational change in the protein, allowing it to oligomerize. It then recruits the adaptor protein ASC, and recruits pro-caspase-1, forming the NLRP3 inflammasome.^140^ Upon activation, NLRP3 facilitates the conversion of procaspase-1, an inactive precursor, to its active form, caspase-1. Caspase-1, in turn, cleaves pro-IL-1β into its mature, bioactive form. Once mature, IL-1β is secreted from the cell and binds to its receptor, IL-1 receptor type I (IL-1RI), on the surface of IVD cells.^139^

The binding of IL-1β to IL-1RI initiates a conformational change in the receptor, allowing the recruitment of the IL-1 receptor accessory protein (IL-1RAcP).^99,141^ Together, IL-1RI and IL-1RAcP form a high-affinity signaling complex that, similarly to the TLR, recruits the MyD88/IRAK/TRAF6 complex.^99,141,142^ TAK1 is then activated and ultimately results in activation of the NF-κB and MAPK signaling pathways.^99^

**Fig. 2:**
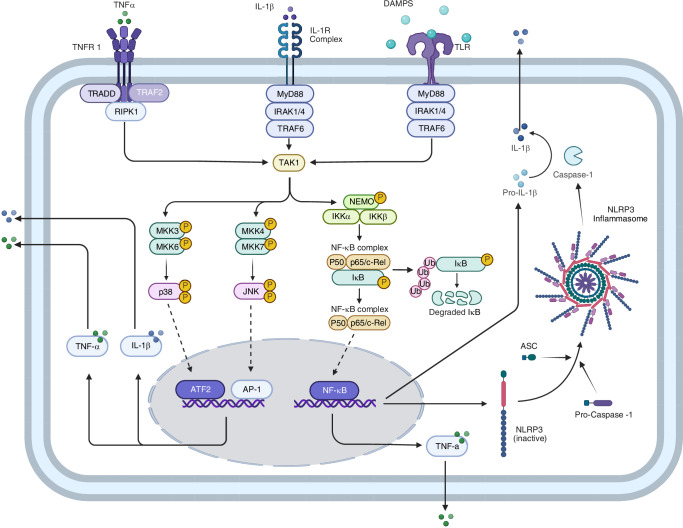
Molecular mechanism of action for IL-1β and TNF-α

Certain DAMPs can also be responsible for upregulating TNF-α via the NF-κB pathway, however, not through activation of the inflammasome. Instead, DAMPs bind to cell surface PRRs such as those of the TLR family or RAGE.^143,144^ Activation of PRRs leads to downstream recruitment and activation of the IKK complex, which is responsible for releasing NF-κB dimers upon phosphorylation. These dimers (typically p50 and p65), are then able to translocate into the nucleus and bind to the promoter region of the TNF-α gene, thus upregulating TNF-α production.^145^ TNF-α is then secreted out of the cell, where it can bind to surface receptors and activate other signaling pathways that contribute to inflammation.^135,146^ The chronic activation of these pathways leads to progressive ECM destruction, increased cellular senescence, disc cell death, and impairment of biomechanical function, all of which are characteristic features of IVDD (Fig. 2).^147,148^

Key mediators of IVDD, namely IL-1β and TNF-α, are typically produced by immune cells but are also secreted by IVD cells.^99^ Low levels of these cytokines are present in non-degenerative disc cells, indicating their role in tissue reconstruction. However, higher levels of expression are observed in IVDD, correlating positively with age and the degree of disc degeneration.^125^ Notably, TNF-α is more abundant in NP cells compared to AF cells, and receptors like TNFR1, TNFR2, TACE, and IL-1RI are expressed in NP tissues.^149^ Overexpression of IL-1β and TNF-α in degenerated IVDs may be a causative factor in IVDD.^99^

Given the central role of IL-1β and TNF-α in the inflammatory processes of IVDD, targeting these cytokines with specific inhibitors or regulatory molecules offers potential therapeutic benefits.^99^ Anti-IL-1 and anti-TNF therapies may alleviate disc degeneration and improve patient outcomes by reducing inflammation and slowing down the degenerative process.^99^

Angiogenesis, the formation of new blood vessels, is a notable characteristic of symptomatic IVDD.^43^ Typically avascular, IVDs become sites of increased vascularization during degeneration, which is considered critical for symptomatic IVDD.^150^ During inflammation, a macrophage-related cascade results in the secretion of high levels of pro-inflammatory cytokines, further promoting angiogenesis.^151^ Mechanistically, the hypoxic conditions in degenerating discs lead to the upregulation of hypoxia-inducible factor 1-alpha (HIF-1α), a regulator of angiogenesis that stimulates the transcription of pro-angiogenic factors such as vascular endothelial growth factor (VEGF), fibroblast growth factor (FGF), angiopoietin-1, and platelet-derived growth factor (PDGF).^152,153^ These factors promote endothelial cell proliferation, migration, and differentiation, leading to the formation of new blood vessels.^153^ Endothelial cells in existing vessels respond to the secretion of these angiogenic factors by migrating towards hypoxic, nutrient-deprived areas within the disc, forming endothelial sprouts that eventually establish a network of neovascularization.^154^

The SDF1 (CXCL12) /CXCR4 axis also facilitates angiogenesis in degenerated discs by activating the PI3K/AKT pathway.^155^ SDF1 is a chemokine that is upregulated in response to tissue injury, inflammation, and hypoxia. SDF1 binds to the CXCR4 receptor in endothelial cells, promoting cell migration, proliferation, and survival.^155^ Additionally, SDF1 recruits immune cells such as macrophages and T cells, further contributing to tissue inflammation. Crosstalk between SDF1/CXCR4 and VEGF signaling amplifies angiogenesis.^156^ TNF-α increases the expression of VEGF, IL-8, and bFGF in endothelial cells, promoting angiogenesis.^157^

While angiogenesis can improve nutrient delivery to NPCs and facilitate the phagocytosis of dead cells by immune cells, it also promotes inflammation and may exacerbate disc degeneration.^150^ There are conflicting views on the role of HIF-1 α in IVDD; some believe it worsens the condition, while others argue it helps by improving nutrient supply and immune cell infiltration.^152^ Hypoxia plays an important role in angiogenesis, aiming to balance the activation of the HIF-1α/VEGF axis to maintain cellular function. Overall, the role of angiogenesis in IVDD is complex and multifaceted, involving a delicate balance between beneficial and detrimental effects.

### Neurotrophins and innervation

In a healthy IVD, innervation is predominantly limited to the outer third of the outer AF, with a majority of the disc being aneural. However, in the context of IVDD, the innervation pattern undergoes significant changes, with nerves extending deeper into the disc, particularly into the normally aneural NP. This phenomenon is prominently observed in cases of chronic LBP.^158–160^ Previous studies have identified vascularization of the disc as a pathway for LBP development; however, that does not explain why pain is exhibited only in some degenerated discs.^161^

Neurotrophic factors are defined as a family of biomolecules that support the growth, survival, and differentiation of neurons and encompasses a wide range of subfamilies including neurotrophins, neuropeptides, and TGF-β family factors.^162^

Most well-known of these neurotrophic factors are neurotrophins, which are a small subset of structurally similar neurotrophic factors comprised of NGF, BDNF, NT-3, and NT-4 (Table 1). These essential proteins, well known for their roles in neuronal development, survival, and maintenance, have emerged as key players in disc innervation dynamics. While traditionally associated with nervous tissue, neurotrophins have been found to be expressed in non-neural cells within the disc microenvironment, indicating their broader involvement in tissue homeostasis and response to injury.^163–165^ The exact neurotrophins or factors that induce neural ingrowth into the disc remain unknown, but multiple studies have observed upregulation of neurotrophic factors such as Neural Growth Factor (NGF) and Brain-Derived Neurotrophic Factor (BDNF) in degenerated IVD tissues and LBP samples, suggesting that neurotrophins likely play a key role in LBP development.^165,166^

**Table 1:** Low back pain markers

Gene symbol	Gene function	References
BDKRB1	G-protein coupled receptor that binds to bradykinin and is synthesized in pathophysiologic conditions such as inflammation, trauma, burns, shock, and allergy. Ultimately results in chronic and acute inflammation	^29,390^
CGRP (CALCA or CALCB)	Neuropeptide that is involved with pain signaling, vasodilation, neurogenic inflammation, and modulation of autonomic nervous system	^29,128,146,276–280,330,390–395^
	CALCA: accounts for >90% of total CGRP expression in most tissues. Primarily expressed in CNS and PNS	
	CALCB: Mainly expressed in enteric nervous system.	
COMT	Enzyme involved inactivation of catecholamine neurotransmitters transmitters including dopamine, epinephrine and norepinephrine	^29,87,390,396^
IL-6	Pleiotrophic cytokine that plays an important role in inflammation, immune response, etc.	^29,279,330,397–400^
TMEM100	Involved in vascular development	^397^
VEGF-A	Growth factor in PDGF/VEGF family. Heparin-binding protein that induces proliferation and migration of vascular endothelial cells	^401,402^
CD31 (PECAM1)	Protein found in endothelial cell junctions and is likely involved in leukocyte migration, angiogenesis and integrin activation	^195,401,402^
IL-1B	Produced by activated macrophages then activated by caspase 1. important mediator of inflammatory response and other cellular responses such as proliferation, differentiation and apoptosis	^165,330,394,398–402^
IL-17	Pro-inflammatory cytokine produced by T-helper 17 cells	^400,402,403^
TAC1	Precursor protein for Substance P	^146,404,405^
SubP (Substance P)	Neuropeptide associated with pain and noxious stimuli	^280,393,398,404^
NPY	Neuropeptide and neurotransmitter synthesized by GABAergic neurons involved in various physiological and homeostatic processes in both the central and peripheral nervous system	^219,395,406^
BDNF	Neurotrophin that primarily involved in the regulation of growth, maintenance, proliferation, and survival of certain target neurons	^29,165,401,405,407^
NGF	Neurotrophic factor and neuropeptide with nerve growth stimulating activity and the complex is involved in the regulation of growth and the differentiation of sympathetic and certain sensory neurons.	^165,279,330,390,401,405,408^
NGFR (p75NTR)	Non-specific neurotrophin receptor	^195,333,409,410^
NTRK1 (TrkA)	Receptor for NGF. Activation leads to cell differentiation, and may play a role in specifying sensory neuron subtypes	^165,195,333,405,409^
NTRK2 (TrkB)	Receptor for BDNF.	^405,410^
ATF3	Transcription factor involved in physiological stress response. Marker of DRG neuron injury, Androgen signaling repressor	^330,408,411^
TNF	Proinflammatory cytokine mainly secreted by macrophages but can be produced by other cell types.	^128,165,253,279,330,398,400^
TRPV1	Non-selective cation channel activated by noxious stimuli including heat. Thought to function as a transducer of painful thermal stimuli	^146,280,333,392,399,412,413^
TRPV4	A2 + -permeable, nonselective cation channel that is thought to be involved multiple physiological functions. Activated by osmotic, mechanical, chemical, and thermal cues	^399,414,415^
IL-8 (CXCL8)	Cytokine secreted by mononuclear macrophages, neutrophils, eosinophils, T lymphocytes, epithelial cells, and fibroblasts. It functions as a chemotactic factor by guiding the neutrophils to the site of infection	^3,128,399,416,417^
UCHL1 (PGP9.5)	Enzyme involved in hydrolysis of peptide bond at C-terminal glycine of ubiquitin. Specifically expressed in neurons and in cell of the diffuse neuroendocrine system	^276,277,395,401,418^
GAP43	Involved in neuronal growth, development and regeneration. Highly expressed in growth cones. Involved in the formation and strengthening of synapses	^195,330,408^
RAMP1	Required to transport calcitonin-receptor-like receptor (CALCRL) to the plasma membrane	^391^
CALCRL	G-protein coupled receptor related to calcitonin receptor. Associated with RAMPs to produce different receptors. Ex: RAMP1 and CALCRL produces a CGRP receptor	^391,419^
NK1R (TAC1R or SPR)	G-protein coupled receptor found in the nervous system activated by substance p	^398,405^
NTN1	Thought to be involved in axon guidance and cell migration during development	^224,420^
SCN8A (Nav1.6)	Sodium channel found in various tissues including neurons, heart and glial cells	^421,422^
SCN9A (Nav1.7)	Sodium channel found in various neural tissues	^278,281,423^
SCN10A (Nav1.8)	Sodium channel found in DRG and associated with pain and neuropsychiatric disorders	^418,423^
AIF1 (IBA1)	Induced by cytokines and interferons and protein that binds actin and calcium. induced by cytokines and interferon and may promote macrophage activation and growth of vascular smooth muscle cells and T-lymphocytes	^235,279,395^
GFAP	Major intermediate filaments found in mature astrocytes. Marker used to distinguish astrocytes from other glial cells	^128,235,279,282,394,395^

Neurotrophins exert their effects by binding to receptors on the surface of neurons, namely the Tropomyosin Receptor Kinase (Trk) receptors and the p75 neurotrophin receptor, initiating signaling pathways that promote neuronal survival, growth, and differentiation.^160,167^ Interestingly, neurotrophins can bind to both Trk receptors and the p75 neurotrophin receptors, which fall into two completely distinct classes of receptors. First, Trk class receptors, including TrkA (for NGF), TrkB (for BDNF, NT-4, and NT-5), and TrkC (for NT-3), are high-affinity receptors that, upon neurotrophin binding, undergo dimerization and autophosphorylation of their tyrosine kinase domains.^168–171^ This activation initiates downstream signaling cascades, such as the Ras/MAPK pathway and the PI3K/Akt pathway, eventually leading to axon outgrowth, branching, and guidance toward the source of neurotrophins in the target tissue.^168–174^

Additionally, neurotrophin signaling involves the p75 neurotrophin receptor (p75NTR), a low-affinity receptor capable of binding all neurotrophins with lower specificity but at a broader range. This receptor can act independently or in conjunction with Trk.^175–178^ Unlike Trk receptors, p75NTR can modulate both independent and cooperative signaling with Trk receptors, influencing pathways such as Akt signaling and cellular responses such as apoptosis.^179^ NGF and BDNF are two of the most common neurotrophins studied in IVDD and LBP and are involved in the growth, development, maintenance, and survival of neurons. In the context of IVDD and LBP, both are produced by stressed NPCs and immune cells within the disc and act as a chemotactic factor that guides the outgrowth of axons from peripheral neurons toward the target tissue.^174,180–183^ In addition to their role in promoting the outgrowth of axons, NGF and BDNF are also involved in the synthesis of neurotransmitters and neuropeptides, including substance P and CGRP.^174,181,182^ Both NGF and BDNF trigger a complex cascade that results in transcriptional changes, axonal sprouting, and neuronal sensitization, contributing to the progression of pain observed in IVDD.^184–186^

NGF initiates intracellular signaling pathways by binding to its high-affinity receptor TrkA, which is prominently expressed on nociceptive nerve fibers within degenerated intervertebral discs. Upon NGF binding, TrkA receptors dimerize and undergo autophosphorylation of their tyrosine kinase domains, creating docking sites for adaptor proteins like Src homology 2 domain containing (Shc) and growth factor receptor-bound protein2 (Grb2).^187^ This event leads to the activation of the MAPK/ERK pathway, where Grb2 recruits son of sevenless (SOS) to activate Ras. Activated Ras triggers a phosphorylation cascade involving RAF, MEK, and ultimately ERK. Phosphorylated ERK translocates to the nucleus where it phosphorylates transcription factors such as cAMP response element-binding protein (CREB), thereby regulating gene expression related to neuronal survival, growth, and plasticity.^188^

Concurrently, NGF-TrkA binding activates phosphoinositide 3-kinase (PI3K), which phosphorylates phosphatidylinositol 4,5-bisphosphate (PIP2) to produce phosphatidylinositol (3,4,5)-trisphosphate (PIP3). PIP3 recruits and activates Akt (protein kinase B) at the plasma membrane, leading to downstream effects that promote cell survival, inhibit apoptosis, and regulate protein synthesis.^189,190^ Additionally, NGF-TrkA signaling activates phospholipase C-gamma (PLC-γ), which hydrolyzes PIP2 to generate inositol trisphosphate (IP3) and diacylglycerol (DAG).^191^ IP3 induces calcium release from intracellular stores, modulating cellular processes including synaptic transmission, while DAG activates protein kinase C (PKC), involved in various signal transduction pathways. Together, these pathways regulate genes involved in neuronal survival, axonal sprouting, and nerve sensitization.^192,193^ Pathway activation promotes axonal growth and branching by increasing the expression of cytoskeletal proteins, enhancing local protein production, and regulating microtubule and actin assembly.^192^ Nerve sensitization is also promoted through an increase in expression and traffic of ion channels such as TRPV1 to the cell membrane and promoting the release of neuropeptides that amplify the pain signal.^192^

NGF has been extensively studied as a possible cause of innervation due to various studies identifying it as being upregulated in degenerated IVD tissue.^166,194^ Interestingly, a study by Freemont et al. found that LBP-causing discs had microvascular vessels within the inner AF and NP that produced NGFβ, while non-painful discs did not. This finding aligns with our hypothesis that vascular ingrowth causes neural ingrowth.^195^ Interestingly, Freemont determined that all vessels expressing NGFβ originated from the endplate, suggesting a possible role for the endplate in innervation and LBP.^195^

**Fig. 3:**
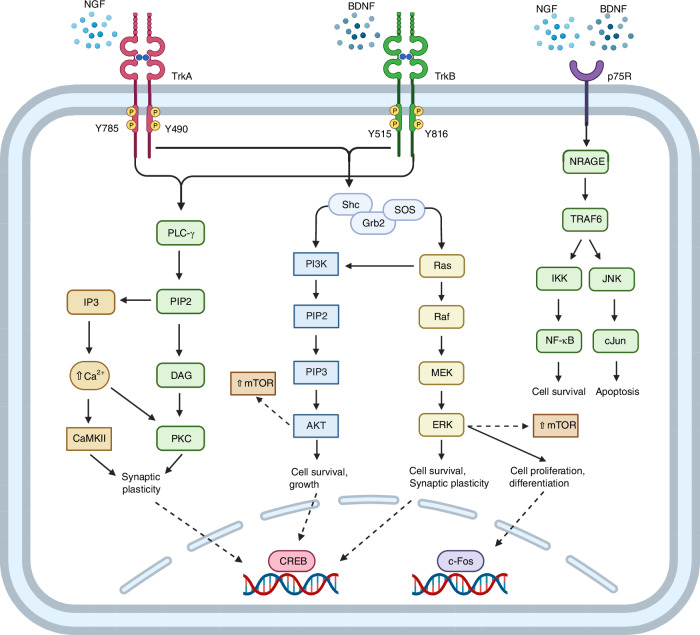
Molecular mechanism of action for NGF and BDNF

Similarly to NGF, BDNF is a neurotrophin known for its ability to induce nerve growth and has been found to be expressed by IVD cells across all stages of degeneration.^165,166^ Research indicates that stimulation with pro-inflammatory cytokines such as IL-1β can induce BDNF secretion by IVD cells. Once secreted, BDNF binds to its receptor TrkB, leading to dimerization and autophosphorylation, thus activating the receptor.^165^ The activated TrkB receptor then engages several downstream signaling pathways, including PLC-γ, PI3K, MAPK, and ERK. Specifically, TrkB activates PLC-γ via phosphorylation of the Y816 residue, increasing intracellular calcium levels and activating calcium/calmodulin-dependent protein kinase II (CaMKII).^196^ Consequently, this activates the CREB transcription factor, which promotes gene expression related to neuronal survival and plasticity.^197^ Furthermore, TrkB activation also stimulates the PI3K pathway by recruiting Shc, which activates the Grb2/SOS complex.^198^ This leads to the activation of downstream molecules such as Ras and Akt, promoting cell survival and growth by inhibiting apoptotic pathways.^198^ In the MAPK pathway, activated Ras initiates a kinase cascade involving Raf, MEK, and ERK. ERK then translocates to the nucleus, activating transcription factors that support neuronal differentiation and survival.^199^ The activation of these pathways culminates in the activation of transcription factors such as CREB and mTOR, which drive the expression of genes and the translation of proteins essential for neuronal growth, differentiation, and synaptic plasticity (Fig. 3).^200^

In addition to their role in producing cytokines, immune cells such as T-cells, B-cells, and monocytes have been found to produce neurotrophins, including NGF, BDNF, NT-4 and NT-5, as well as their receptors, TrkA, TrkB, and TrkC.^201^ These neurotrophins are produced downstream of TLRs such as TLR2 and TLR4, which activate inflammatory pathways like IL-1β and NF-κB.^202^ Stress on NPCs has been suggested as an alternative pathway for the upregulation of neurotrophins within the IVD. Multiple studies have identified NPCs as being able to produce neurotrophins and induce nociceptor growth and innervation in vitro.^3,183,203^ A recent study by Jiang et al. found that compared to non-stressed NPCs, stressed NPCs, that is, NPCs exposed to pro-inflammatory conditions induced significantly more axon ingrowth in vitro.^3^ These results suggest that NPC stress likely plays a role in inducing innervation of the disc, whether by expression of neurotrophins and neo-innervation factors or by secretion of protective factors that prevent innervation from occurring. Indeed, both neurotrophic and pain factors have been found to be positively regulated by cytokines such as IL-1, IL-6, and TNF-α, which are commonly found to be upregulated during IVDD.^166^ These findings suggest that IVDD and, in turn, expression of pro-inflammatory cytokines may induce innervation and pain.^166^

Another commonly studied group of neurotrophic factors are GDNF family ligands (GFLs) which is composed of GDNF as well as neurturin (NRTN), artemin (ARTN), persephin (PSPN). Due to its structure, this family falls into the TGF-β superfamily which includes non-traditionally neurotrophic factor subfamilies such as TGF-β, BMPs, activins, and inhibin. Regarding discogenic LBP, GFLs are the most studied factors and have been linked to neurite outgrowth, neuroprotection, neuronal survival, etc.^204^ All four of these ligands can exert their effects though three different receptor systems. The first and most well-understood is through the GDNF family receptor alpha (GFRα). GFLs bind with the four characterized GFRα, however, each GFL binds one of the GFRα as its highest affinity receptor.^204^ Specifically, GFRα1 interacts with GDNF, GFRα2 with NRTN, GFRα3 with ARTN, and GFRα4 with PSPN.^204^ Once the GFLs bind to one of the GFRα, it forms a complex with tyrosine kinase Rearranged During Transfection (RET), which is then able to activate RET via dimerization and transphosphorylation of its tyrosine residues. The activated RET then induces a downstream signaling cascade through the PI3K/Akt and MAPK pathways. Another pathway used by GFLs is low affinity binding with NCAM, which increases the binding affinity of the GFLs with GFRα1.^205^ The GFL-NCAM- GFRα1 complex can then activate Src-like kinase Fyn and focal adhesion kinase FAK and induce downstream signaling.^205^ Finally, GFLs can also interact with syndecan-3, which is a heparin sulfate proteoglycan, in the absence of co-receptors.^206^ Once formed, GFL-syndecan-3 complexes can act as both co-receptors, bringing the GFL to GFRα and inducing downstream RET signaling, and independently, directly activating Src-dependent signaling.^205^ Though GDNF has been long shown to be expressed in degenerated IVD, recent studies have also found GFLs NRTN, ARTN, and PSPN expressed in cells derived from degenerated IVD suggesting they may play a role in disc innervation and LBP development.^207,208^ Furthermore, stimulation with IL-1β was found to upregulate expression of these ligands and their co-receptors both at the mRNA and protein level.^208^

In addition to neurotrophic factors, neuropeptides are small proteinaceous substance that function as neurotransmitters and neuromodulators, influencing neurotropic activity.^209^ While they are mainly associated with neurons, some neuropeptides have also been expressed in non-neural cells, including within the IVD.^166,210^ These neuropeptides bind to G-protein coupled receptors (GPCRs) with high affinity and, unlike classic neurotransmitters, are active at much lower concentrations and have slower reuptake by synapses, allowing them to exert prolonged effects through various downstream pathways (Table 1).

One of the most well-known neuropeptides associated with pain is Substance P (SubP). SubP is not only endogenously expressed by IVD cells but is also found in a greater percentage of cells in degenerated IVD samples (Table 1).^166,210^ SubP is derived from the alternative splicing of the preprotachykinin A (TAC1) gene and functions both as a neurotransmitter and neuromodulator. Typically stored in vesicles within neurons, SubP is released in response to various stimuli, including inflammation.^211^ Studies have also found that SubP is produced by IVD cells themselves, particularly under conditions of degeneration.^212^ Once released, SubP binds to one of four neurokinin receptors, with the highest affinity for the neurokinin-1 receptor (NK1R).^213^ The binding of SubP to NK1R activates multiple pathways, including the IP3/DAG pathway and the cAMP pathway, leading to various cellular responses.^213^ Additionally, SubP can induce cytokine production and, in conjunction with nitric oxide, promote vasodilation.^214^ High levels of SubP have been observed in the spinal cords of rats with chronic pain, highlighting its role in neuroinflammation.

Another neuropeptide commonly associated with pain and nociception is Calcitonin Gene-Related Protein (CGRP; Table 1).^215^ CGRP exists in two major isoforms: CALCA, mainly found in the central and peripheral nervous systems, and CALCB, primarily expressed in the enteric nervous system.^215^ Despite their structural similarities, these isoforms have distinct biological functions.^215^ CGRP’s receptor complex requires three subunits for optimal function: the calcitonin receptor-like receptor (CALCRL), a GPCR capable of binding any calcitonin family ligand; the receptor activity-modifying protein 1 (RAMP1), which is CGRP-specific; and the receptor component protein (RCP), which facilitates coupling of the CGRP-CALCRL-RAMP1 complex with downstream signaling pathways, including cAMP.^215^ Activation of this receptor complex leads to an increase in intracellular cAMP, which in turn activates various targets involved in pain signaling and vasodilation.^215^

SubP and CGRP are often co-released and co-localized in neural cells, but they use different receptors—SubP through NK1R and CGRP through the CGRP receptor complex.^216^ Both SubP and CGRP are upregulated in response to pro-inflammatory cytokines like IL-1β and TNF-α, suggesting a role in the inflammatory processes associated with IVDD.^216^ Additionally, neuropeptide Y (NPY) is another neuropeptide that plays a role in nociception and pain modulation.^217^ NPY acts through GPCRs, particularly the Y2 receptor (Y2R), which has been found to be upregulated in degenerated human IVD tissue, indicating a potential role in disc pathology.^218,219^

Transient Receptor Potential (TRP) ion channels are another group of receptors implicated in pain and nociception within the IVD.^220^ The TRP channel family is diverse, responding to various stimuli such as temperature, mechanical forces, and osmotic pressure.^220^ Although TRP channels are primarily associated with neurons, they have also been found in IVD cells, with higher expression levels in degenerated discs.^220^ Surprisingly, 26 of the 28 TRP channels expressed in humans have been found to be expressed by IVD at the mRNA level suggesting these receptors may play a greater role in IVD homeostasis and that previously predicted.^221^

Of the TRP family, TRPV1 and TRPV4 have been directly linked to low back pain (LBP). TRPV1, mainly expressed in nociceptive neurons, is activated by noxious stimuli like low pH and inflammatory mediators, both prevalent in degenerating IVDs. (Table 1). In contrast, TRPV4 is more widely expressed and is involved in detecting osmotic changes and mechanical stimuli, which are crucial for IVD homeostasis.^221^ It plays an important role in detecting osmotic changes and mechanical stimuli, which are both important measures for IVD homeostasis. Indeed, studies into the function of TRPV4 in the IVD have found that activation of the channel in IVD derived cells has been linked to increased production of IL-6.^222,223^ Activation of TRPV1 and TRPV4 channels leads to an influx of calcium ions (Ca^2+^), triggering action potentials and transmitting pain signals to the central nervous system.

In addition to neuropeptides and ion channels, other proteins like Netrin-1 (NTN1), an axon guidance molecule, have been implicated in LBP (Table 1). NTN1, expressed by degenerated nucleus pulposus cells, has been shown to promote neuronal outgrowth, suggesting a role in pathological innervation within the disc.^224^ Moreover, the highly innervated endplate, specifically the bone marrow and periosteum, may also contribute to pathological innervation, further linking these molecular mechanisms to the development of pain in IVDD.

### Peripheral and central sensitization

Over time, as the LBP transitions from acute to chronic, sensitization of both the peripheral and central nervous systems occur.^225^ Sensitization induces neuroplastic changes, recruiting non-nociceptive pathways. This recruitment includes the involvement of low-threshold mechanoreceptor inputs to pain pathways and the conversion of nociceptive-specific neurons to wide dynamic range neurons that can respond to both painful and non-painful stimuli.^226^

Recent studies have identified sensitization of the peripheral and central nervous systems as playing a significant role in chronic LBP.^3^ Peripheral sensitization refers to neuropathic pain mediated by amplified nociceptor stimulation at normally sub-threshold levels for pain.^227^ In discogenic LBP, inflammatory cells respond to ECM degradation and destruction of the disc structure via the release of cytokines such as IL-1β, IL-1α, and TNF-α that increase the synthesis of NGF.^228^ Elevated levels of neurotrophins, like BDNF and NGF, have shown evidence of leading to the ingrowth of sympathetic nerve fibers that penetrate through the outer AF and endplates into the inner AF and NP.^165,167^ These infiltrating nerves express substance P, TrkA receptors, and increase the production of BDNF with increasing IVDD.^165,167^ Furthermore, as nerves penetrate the IVD, cytokines released into the disc space activate nociceptors, increase inflammatory marker receptors, and upregulate the activation of Ca_v_2.2 voltage-gated calcium channels that cause calcium influx into the cell and may play a key role in mechanoresponse.^165,167,229^ Inflammatory factors such as IL-1β, IL-6, and TNF-α also can influence sodium channels such as TRPA1, TRPV1, Na_V_1.7, Na_V_1.8, and Na_V_1.9.^230^ Over time, an increase in neural discharge results in sensitization of ingrowing sympathetic nerves to fire at previously sub-threshold levels. It is believed that these neurons are important in pain propagation and the feeling of chronic LBP, as observed in a rat model by Murata et al. which demonstrated that when ingrowing sympathetic nerves were ablated, there was a decrease in pain response.^231^ The barrage of pain signaling induces progressive amplification from consistent input, also known as “wind-up,” resulting in an increase in synaptic efficacy and neuronal excitability.^232^

Central sensitization, however, refers to changes in the properties of CNS nociceptors to respond to stimuli that previously would not have caused a response, increasing the potential for hyperalgesia and allodynia.^233^ Excessive nociceptive signals from the peripheral nervous system within the dorsal horn of the spinal cord can result in transcriptional and translational changes in second order neurons, thus causing lingering hyperexcitability known as central sensitivity. In addition, inhibitory controls also tend to be affected, thus creating an imbalance between excitation and relaxation.^230^

In addition, neuroinflammation has been identified as also playing a role in chronic pain development. During acute injury, local macrophages, glial cells, and injured neurons signal immune cells to release pro-inflammatory cytokines, such as IL-1β, IL-6, IL-12, and TNF-α, inducing neuroinflammation.^234^ The influx of inflammatory markers at the site of injury leads to the discharge of nociceptors that propagate the signals up to the spinal cord.^234^ However, in a recent study on neuroinflammation in a rat model of IVDD, researcher found that neuroinflammation may last for far longer than initially predicted with rats exhibiting increased satellite glial cells and active macrophages up to eight weeks post injury.^235^ Furthermore, signs of neuroinflammation were identified not only in the DRGs but also in the dorsal horn of the spinal cord.^235^

## Modeling discogenic pain

### Ex vivo models using organ cultures

Cell monoculture is the most basic and long-standing model for studying IVDD and has played a vital role in our understanding (Table 2). Like many cell types, studies on IVD cells have found that the method of cell culture significantly impacts cell morphology, gene expression, and cell behavior. Indeed, multiple studies comparing cells in situ, monolayer, and 3D culture of IVD-derived cells have identified significant differences in cell morphology.^236–239^ In vivo, NPCs were found to have a rounded shape and be surrounded by a distinct capsule, while the cells of the AF took on an elongated appearance following the direction of the collagen fibers of the disc.^238^ Studies examining IVD cells in vitro found that, initially, the morphology observed in native discs is preserved after cell isolation and seeding, with AFCs maintaining a spindle-shaped fibroblast-like morphology and NPCs adopting a polygonal appearance.^237^ However, morphological changes were found to occur shortly after initial seeding, with some studies observing changes as soon as three days in monolayer culture.^240^ Gene expression analysis studies of IVD-derived cells in monolayer culture over time demonstrate a loss of aggrecan, collagen I, and collagen II expression, resembling patterns observed in articular cartilage monolayer culture.^237,239^

**Table 2:** In vitro models of IVD degeneration and low back pain

In vitro model	Culture method	Cells / tissues	Pros	Cons	References
Monolayer culture	Various substrates (Polystyrene, PDMS)	All types of IVD derived cells (NP, AF, etc)	Simple to use, inexpensive, easy to manipulate cellular environment	Cells quickly lose in vivo morphology once seeded, lack of ECM and mechanical stimuli may affect cell behavior	^236,237,424^
Microfluidics	Tissue	Whole disc	Most representative of disc tissue, good for nutrient exchange and perfusion studies	Difficult to monitor inside of disc, often requires specialized equipment	^241,245^
	Monolayer	Multi-cell type culture (ex: AF, NP, Neural, EC)	Ideal for cellular crosstalk studies, allowed for precise control of microenvironment	Susceptible to seeding effects and technical difficulties (ex: air bubbles), more expensive than traditional monolayer culture, often requires additional specialized equipment	^3,244,246,247^
3D culture	Hydrogel	All types of IVD derived cells (NP, AF, etc)	Preserves 3D environment, supports native cell-matrix interactions	Requires specific culture conditions, longer-term viability limitations	^166,183,210,237–239^
	Woven scaffold	AF, IA, NP	Provides scaffold-based culture system	May not fully replicate native disc matrix, cells may not fully integrate into scaffold system	^239^

Given that IVDD and LBP are chronic diseases, experiments involving long-term culture are required, making monoculture non-ideal for long term study. To address these limitations, researchers have turned to 3D culture of cells, which has been found to preserve some of the features lost in monolayer culture. Compared to monolayer culture, 3D culture has been shown to significantly improve cell viability, matrix production, and phenotypic stability. However, these models still have their limitations and may not fully recapitulate the complex microenvironment of the native IVD. Various materials, including alginate and collagen scaffolds, have been employed to create 3D environments.

### Organ-on-chip systems

More recently, the development of organ-on-chip systems and microfluidic chips has become increasingly used and is an excellent method for small-scale modeling and studying cellular crosstalk (Table 2). Given that IVDD is a chronic disease, and that IVD-derived cell morphology and functionality are rapidly lost in monolayer culture, strides have been made to improve the long-term culture of the IVD and their derived cells. Various versions of IVD-on-chip utilizing microfluidic technology have been developed to provide sufficient nutrient transport and waste removal.

Dai et al. developed a “disc on chip” microfluidic chip, enabling long-term culture of whole mouse IVD in vitro.^241^ Unlike static culture methods, the IVD cultured in the “disc-on-chip” system retained matrix organization, cellular phenotype, cell viability, and overall structural integrity comparable to native discs for 10 to 21 days post-seeding.^241^ Various studies have identified that mechanical stimulation is essential for maintaining IVD health and function.^242,243^ McKinley et al. developed a multiaxial strain AFC-on-chip system combining microfluidic technology with multiaxial strain to allow for long-term study of strain on IVDD.^244^ More recently, Xie et al. combined the developments of McKinley and Dai to create a mouse IVD mechanical loading chip system with dynamic media flow.^245^ The combination of a continuous nutrient supply plus appropriate mechanical loading resulted in structural integrity, collagen breakdown and alignment, catabolic enzyme activity, and cell alignment comparable to native IVD and significantly improved compared to static culture up to 21 days.^245^ Though promising, these systems still lack the ability to preserve disc morphology for long-term study and thus require additional optimization.

In addition to enhancing long-term culture of IVD cells in vitro, microfluidic organ-on-chip systems have emerged as valuable tools for studying cellular crosstalk within the IVD microenvironment. These systems offer a unique advantage for investigating cellular interactions by fluidically isolating cells while enabling their interaction.

Given the recognized role of immune involvement in the progression and development of pain in IVDD, researchers have utilized microfluidic platforms to delve into these aspects. Son et al. developed an NP-monocyte chip to investigate monocyte extravasation when exposed to IL-1β-stimulated NP cells.^246^

More recently, NP cells have been identified as a potential source of factors involved in neurogenesis and initiating innervation of the disc. Jiang et al. developed an iPSC-derived nociceptor-nucleus pulposus cell microfluidics model using the Xona chip (Xona Microfluidics) which studied nociceptor-NPC interaction.^3^ Similarly, Zheng et al. utilized standard neuron device (Xona Microfluidics) to explore innervation in response to conditioned media from degenerated NP cells.^224^ Hwang et al. developed a microfluidics chip designed to examine the paracrine interaction between AFC, NPC, and endothelial cells and neurons via a microfluidic gradient system.^247,248^

### Animal models

Animal models serve as invaluable tools for studying pain, including discogenic pain, as it is currently impossible to fully recapitulate the pain signaling process in vitro. A diverse array of animal models, ranging from small organisms like zebrafish, mice, and rats to larger mammals such as pigs, dogs, and sheep, have been developed to investigate IVDD (Table 3).^29,30,249–273^ However, very few specifically study discogenic pain. Studies focusing on discogenic pain in animals are almost exclusively performed in mice and rats.^272–275^ Several factors contribute to the predominance of murine models in discogenic pain research. Firstly, a rich body of literature exists on IVDD, pain mechanisms, and overall anatomy in murine models, along with a plethora of well-established pain tests, making them a reliable and widely utilized model for discogenic pain research. Additionally, their small size and ease of handling and training make them ideal for conducting biobehavioral pain assessments. Moreover, murine models are cost-effective to procure, house, and maintain compared to larger animals. Furthermore, the availability of diverse pre-existing genotypes and the ease of generating new genetic models make murine models ideal for pain research. In murine models, disc puncture method of IVDD induction is the preferred choice in the field due to its ease of induction, low cost and procedure simplicity. This model most commonly targets the lumbar or caudal discs. Some labs, like ours, prefer the lumbar discs to maximize clinical relevance, while others opt for the caudal levels due to their greater accessibility.^78,79,224,270–293^ Other methods of IVDD induction have been developed, including surgical induction, injection of noxious substances such as TNF-α or Freud’s adjuvant, and the use of custom spine-stretching apparatuses, however, these alternatives are less popular due to the simplicity and effectiveness of the needle puncture method.^3,294–299^

**Table 3:** Animal models of IVD degeneration and low back pain

Model size	Species	Induction method	Pros	Cons	References
Small	Zebrafish	Spontaneous	Genetically modifiable, easy to house, inexpensive, ideal for IVD development studies	No BBT available, limited applicability to humans, longer time to IVDD onset.	^249^
	Mouse	Disc puncture	Inexpensive, easy to house, well-established model, widely validated, wide range of robust and validated BBTs available, less invasive	Retains notochordal cells into adulthood, rapid healing limits degeneration severity, significantly different biomechanical and diffusion properties compared to human IVD, puncture injury can be inconsistent	^270,271,276,277,290–293^
		Transgenic mice (ex: SPARC^-/-^, miR-183 KO, etc.)	IVDD often occurs spontaneously, easy to house, genetic modification available, wide range of robust and validated BBTs available	More expensive than wild-type mice, often requires colony investment and genotype tracking, often has a long degeneration onset	^128,313,394,425–428^
		Tail looping/tail suspension	Non-invasive, cost-effective, mimics postural and mechanical load-related degeneration	slow degeneration onset, variability in individual responses, may induce additional systemic effects (e.g., circulation changes, muscle atrophy)	^299^
Medium	Rat (SD or Wister)	Disc puncture	Inexpensive, easy to house, well-established model, widely validated, larger than mouse, wide range of robust and validated BBTs available, less invasive than surgical methods	Retains notochordal cells, different biomechanics and diffusion vs. humans, puncture technique and needle size influence variability in degeneration progression	^78,79,224,272–275,278–289^
		Injection	Models cell-induced pain, may better represent pain-related pathology, easy to house, inexpensive, wide range of robust and validated BBTs available, less invasive than surgical methods	Retains notochordal cells, technically challenging procedure, injection technique and needle size influence variability in degeneration progression	^3,294,295^
		Disc puncture with injection	Easy to house, inexpensive, wide range of robust and validated BBTs available, pain and/or degeneration can be induced independently, less invasive than surgical methods	Injected solutions such as cytokines significantly exceed natural conditions, technique and needle size influence variability in degeneration progression	^316,397^
		Spinal attachment unit	More natural induction of degeneration, easy to house, inexpensive, wide range of robust and validated BBTs available, minimal invasive	Requires specialized surgical expertise and equipment	^298^
		Surgically induced disc injury (ex: Nuclectomy)	Fast and severe degenerative phenotype, difficult to control Easy to house, inexpensive, wide range of robust and validated BBTs available,	Technically challenging, less accurate representation of human degeneration phenotype, invasive, increased risk of inflammation and off-target tissue damage affecting pain outcomes	^296,297^
	Rabbit	Injection	Larger disc size compared to rodents, limited BBT methods available	Expensive, requires larger housing compared to murine models, does not fully replicate natural disease progression, injection technique and needle size influence variability in degeneration progression	^429^
		Disc puncture with injection	Larger disc size compared to rodents, limited BBT methods available	Requires skilled surgical techniques, potential variability in injury severity, puncture/injection technique and needle size influence variability in degeneration progression	^250,251^
		Disc Puncture	Established model for degeneration studies, larger disc size compared to rodents, easier for surgical manipulation, limited BBT methods available	Requires skilled surgical techniques, Puncture technique and needle size influence variability in degeneration progression	^250^^,251^^,^^430–432^
Large	Pig	Disc puncture	Well-established model for degeneration studies, BBTs exist in other fields of study, more biomechanically accurate than rodents, minimally invasive	Expensive, difficult to house, needs robust preliminary data prior to using model, retains notochordal cells into adulthood, expert required to perform BBT and analysis, puncture technique and needle size influence variability in degeneration progression	^29,30,433–437^
		Surgically induced disc injury	Well-established model for degeneration studies, BBTs exist in other fields of study, more biomechanically accurate than rodents, controlled and reproducible degeneration, allows for precise lesion creation, severe fast degeneration onset	Expensive, difficult to house, needs robust preliminary data prior to using model, retains notochordal cells into adulthood, expert required to perform BBT and analysis, minimal representative of natural development of IVDD, increased risk of inflammation and off-target tissue damage affecting pain outcomes	^438–442^
		Endplate injury	Mimics early stage IVDD, more physiologically relevant, minimal damage to disc structure	Specialized equipment required to create precise endplate lesions, injury severity can be difficult to standardize across subjects, may, may cause unintended bone remodeling, may take longer for IVDD-related pain behaviors to manifest compared to other models	^436,443,444^
	Goat	Disc puncture	Well-established model for degeneration studies, BBTs exist in other fields of study, more biomechanically accurate than rodents, BBT methods available in other fields, minimally invasive	Expensive, hard to house, needs robust preliminary data prior to using model, expert required to perform BBT and analysis	^252–255^
		Chondrotinase ABC injection	Well-established model for degeneration studies, BBTs exist in other fields of study, more biomechanically accurate than rodents, BBT methods available in other fields, minimally invasive	Enzymatic digestion of disc non-representative of disease development, expert required to perform BBT and analysis	^445–450^
	Sheep (various breeds)	Surgically induced disc injury	Large disc size, Controlled and reproducible degeneration, allows for precise lesion creation, severe fast degeneration onset	Expensive, difficult to house, needs robust preliminary data prior to using model, limited BBT methods available in other fields, minimal representative of natural development of IVDD, invasive, increased risk of inflammation and off-target tissue damage affecting pain outcomes	^256–262^
		Disc puncture	Large disc size, severity can be somewhat controlled, minimally invasive	Expensive, difficult to house, needs robust preliminary data prior to using model, limited BBT methods available in other fields, moderate representation of natural development of IVDD	^451,452^
		Enzymatic injection (ex: Chondrotinase ABC injection)	Level of degeneration can be precisely controlled, minimally invasive	Expensive, difficult to house, needs robust preliminary data prior to using model, limited BBT methods available in other fields, enzymatic degradation does not replicate full biochemical complexity of IVDD	^453,454^
	Dog	Spontaneous	Naturally occurring IVDD, closer disc structure and degeneration process to humans	Expensive, difficult to house, slow degeneration onset, high variability in disease presentation	^263–266^
		Disc Puncture	Faster induction of IVDD, degeneration severity can be somewhat controlled	Requires specialized surgical expertise, variability in healing response	^455–459^
		Compressive loading	Mimics mechanical stress-related degeneration, non-invasive	Requires prolonged study duration, degeneration rate may be inconsistent	^460,461^
		Surgically induced disc injury	Controlled and reproducible degeneration, allows for precise lesion creation	Invasive, requires specialized surgical expertise, may not fully mimic natural IVDD progression, increased risk of inflammation and off-target tissue damage affecting pain outcomes	^462^
	Non-human primates	Spontaneous	Most similar to human IVDD, cellular and biomechanical similarity to humans, BBTs available	Expensive, difficult to house, requires extensive and robust positive preliminary data prior to using model, ethically challenging, longer time to IVDD onset, expert required to perform BBT and analysis	^267–269^
		Surgically induced disc injury (nucleotomy, discectomy, etc.)	Fast and severe degeneration induction compared to spontaneous, better mimics human pathology than small animal models, BBTs available	Expensive, limited availability, ethical concerns, technically challenging, less accurate representation of degeneration seen in humans, limited data on species-specific responses, expert required to perform BBT and analysis, invasive, increased risk of inflammation and off-target tissue damage affecting pain outcomes	^289,463^
		Injection (ex: saline, bleomycin, etc.)	Fast and severe degeneration induction compared to spontaneous, better mimics human pathology than small animal models, BBTs available, minimally invasive	Expensive, limited availability, ethical concerns, technically challenging, less accurate representation of degeneration seen in humans, limited data on species-specific responses, expert required to perform BBT and analysis	^464^
		Surgical plus injection (ex: Annulotomy + collagenase, etc.)	Fast and severe degeneration induction compared to spontaneous, better mimics human pathology than small animal models, BBTs available	Expensive, limited availability, ethical concerns, technically challenging, less accurate representation of degeneration seen in humans, limited data on species-specific responses, expert required to perform BBT and analysis, invasive, increased risk of inflammation and off-target tissue damage affecting pain outcomes	^465,466^
	Bovine (cow)	Spine motion segment	Excellent for ex vivo biomechanical studies, relatively easy and cost-effective for ex vivo studies	Does not take other anatomical structures into account, cannot be studied long term	^467,468^

While murine models have been instrumental in characterizing discogenic pain, their translational potential to humans is limited. Larger animal models, such as dogs and pigs, hold promise as a translational model due to established BBT methods in other fields of study.^300,301^ Establishing such models in the context of LBP holds promise for advancing our understanding of the etiology of discogenic back pain and expediting the translation of potential treatments from preclinical studies to clinical applications.

## Methods of pain assessment

### Imaging

MR imaging has long been used to identify IVDD; however, it remains unable to identify IVDD pain. Recent developments in MR imaging have allowed for the detection of biochemical changes within the disc, such as changes in pH, GAG content, water content, and more. These changes are present in the IVD before the appearance of morphological changes, making them particularly useful for early detection of IVDD and for diagnosing possible discs exhibiting pain. Advances in MR imaging have resulted in the development of chemical exchange saturation transfer (CEST) imaging and, more recently, qCEST MRI which can noninvasively identify biochemical changes within the disc associated with both degeneration and pain (Table 4).^29–31^

**Table 4:** Imaging methods of assessing LBP development

Imaging	Sequences	Research or clinical use?	Animals imaged?	Pros	Cons	References
MR imaging	T1, T2, MTR, qCEST	Both	Dog, Pig	Non-invasive, gold standard for structural changes	Expensive, current standard of care imaging sequences may not be able to detect pain	^29,31,473–475^
PET/CT	18-Fluorodeoxyglucose, 18-Fluoride	Both	Dog, Pig	Identifies metabolic changes	Expensive, more invasive than other imaging modalities	^476–480^
MRS		Clinical		Non-invasive, can detect biochemical changes	Lack standardization	^481,482^

CEST is an MRI technique that indirectly detects exchangeable protons in the water pool by pre-saturation at different frequency offsets. CEST MRI can image endogenous or exogenous compounds containing protons that exhibit a suitable exchange rate with bulk water. Compared to 1H MR spectroscopy, CEST techniques offer higher in-plane spatial resolution and higher sensitivity for the target molecules.^302^ Recent advances in MRI technologies have allowed researchers to assess pH changes in the body non-invasively.^28,303^ Of note, CEST has been studied to measure pH-dependent MRI signal changes.^304–307^ This technique exploits the pH-sensitive chemical exchange between water protons and solute protons in certain molecules. Previous studies have applied CEST to detect GAG concentration and pH changes in the IVDs of pigs and humans.^303,308^ However, the CEST signal can be affected by other contributing factors, including water relaxation parameters and solute concentration.^309^ To eliminate these confounding factors, qCEST was developed to measure the exchange rate independently from T1- and T2-signals and the solute concentration.^310–312^ In the NP, the hydroxyl proton of GAGs can serve as an endogenous agent for qCEST to measure the pH-sensitive exchange rate with water protons. It has recently been shown that the exchange rate measured using qCEST is closely correlated to pH values found in the IVD.^31^ Though not currently part of the standard of care, the qCEST is a promising method of diagnosing discogenic pain. Unlike other methods, qCEST MRI is non-invasive and can provide definitive, quantitative results, making it ideal for implementation in a clinical setting.

### Biobehavioral testing

In animal models, biobehavioral testing (BBT) has long been the gold standard for pain assessment, as animals cannot effectively communicate their pain to researchers. Due to the labor-intensive nature of these tests and the active involvement required from researchers, development of BBTs assays have primarily been in small, easy to handle models, such as mice and rats. While many tests assess pain by applying stimuli to the hind paw or tail, some have been adapted for other regions, including the back.^313–315^ Generally, these tests fall into four main categories: 1) mechanical hyperalgesia, 2) thermal hyperalgesia, 3) movement evoked hypersensitivity, and 4) spontaneous pain-related behaviors.

#### Tests for mechanical hypersensitivity

Mechanical hypersensitivity tests measure responses to noxious stimuli and include the von Frey (VF), Randall-Selitto (RS), and vocal threshold tests (Table 5). Of all the BBTs used to quantify LBP-induced allodynia and hyperalgesia, the VF is the most common, involving the application of calibrated filaments to the palmar surface of the hind paw.^3,275,313,316–318^ Some modified versions of VF have been developed for application on the lower back.^313–315^ The wide spread use and validity of the test makes it extremely popular for allodynia and hyperalgesia induced allodynia and hyperalgesia, however, due to the variability of animal response to stimuli, experience is required to ensure accurate data collection. Furthermore, some critics argue that interpretation of animal responses is subjective, thus potentially introducing observer bias.^319^ However, using blinded testers and ensuring the same tester conducts all assessments throughout the experiment can substantially mitigate this issue, enhancing consistency and reliability of the data.

**Table 5:** Methods of assessing LBP development

Test type	Test	Clinical or research	Animal model	Advantages	Disadvantages	References
Mechanical hyperalgesia	von Frey	Research	Mouse, Rat	Cheap, widely validated for neuropathic/allodynia assessment	Requires experienced researcher for accurate results, subjective interpretation, limited clinical translation	^275,286,293,294,313,317,318,333,335,336,392,397,404,469–471^
	Randall Selitto	Research	Rat	Provides quantitative measurement of mechanical nociceptive thresholds	Limited to rat models, restraint induced stress may alter pain response	^273,469^
	Pressure algometry / applied pressure vocalization threshold	Research	Mouse, Rat	Quantifies localized tenderness, reliable intra-rater results in clinical studies, relatively cheap	Requires trained examiners for consistency, poor specificity for LBP mechanisms	^275,283,392,410,471^
Thermal hyperalgesia	Tail flick/Hot plate/hot water	Research	Rat	Simple, rapid assessment of thermal nociception,	Tests spinally mediated reflexes, not LBP-specific pathways	^286,294,313,330,339,429^
	Hargreaves	Research	Rat	Focused on radiant heat sensitivity (mimics inflammatory LBP)	Risk of tissue damage, temperature precisely controlled	^286,393,469^
	Acetone/Cold plate/cold water	Research	Mouse, Rat	Detects cold allodynia, fast, relatively easy to perform	Analysis is time-consuming; subjective interpretation, limited clinical application	^275,277,313,318,330,339,397,470^
Movement evoked hypersensitivity	Tail suspension assay	Research	Mouse	Assesses pain-related movement avoidance	Stress confounds pain measurements, no direct validation for lumbar pain	^293,313,330,339,404^
	Grip force	Research	Mouse, rats	Measures functional impairment, cheap	Requires precise force application for accurate results, indirectly assesses LBP	^277,313,318,330,339,404,410,470–472^
	Rotarod	Research	Mouse, Rat	Quantifies dynamic motor deficits	High risk of animal injury, low clinical relevance	^273,293,313,318,330^
	Lateral bending maze	Research	Rat	Requires no handling during testing, highly controlled testing environment	Limited validation in chronic LBP models	^277,313,318,333^
Spontaneous related behaviors	Weight bearing	Research		Detects minute changes in stance and movement	May not be sensitive enough to detect early LBP	^333,335,336^
	Catwalk/gait analysis	Research	Mouse	Captures gait abnormalities	Time consuming analysis	^285,333,338,339^
	Open field	Research	Rat, pig	Easy to perform, assesses general activity	Insensitive to mild LBP, can be confounded by anxiety	^275,313,333,335,341,471,472^

The Randall-Selitto and vocal threshold tests, like the von Frey, assess mechanical hypersensitivity by applying increasing controlled pressure to a localized area, typically the hind paw.^320^ These methods rely on specialized equipment to provide precise quantification of mechanical nociceptive responses. However, compared to the VF, they require more specialized and expensive equipment, making them less commonly used. Additionally, both tests necessitate animal restraint during assessments, which can introduce variability or stress-related effects into the data.^321,322^ However, unlike the multiple filaments used in VF tests, these tests use a single probe which allow for increased standardization between stimulus applications.

#### Tests for thermal hypersensitivity

Pain also induces increased sensitivity to both hot and cold stimuli, making thermal sensitivity assessments an important component of comprehensive pain evaluation. Thermal hypersensitivity tests are typically categorized into heat and cold sensitivity, including tests such as Hargreaves, acetone, hot/cold plate, and hot/cold water (Table 5).

Among these, the acetone test is the most used, involving the application of a drop of acetone onto the plantar surface of the paw to elicit a cooling sensation and a subsequent nocifensive response.^323^ This test is favored for its simplicity, low cost, and rapid administration. However, interpretation of test responses can be subjective and analysis is time-consuming. These limitations can be significantly reduced by providing appropriate tester training and ensuring the same tester conducts all assessments.

Cold water and cold plate assays, like the acetone test, elicits nocifensive responses to cold stimuli, while hot water and hot plate assays use high-temperature stimuli in. These assays measure responses such as tail flicking, paw licking or jumping, reflecting thermal pain sensitivity thresholds.^324–326^

The Hargreaves test, another commonly used assay, measures the latency to paw withdrawal in response to focused heat.^327^ Its simplicity and effectiveness have contributed to its widespread adoption; however, the lack of precise temperature control can lead to variability in results. Additionally, inconsistent heat application increases the risk of tissue injury at the site of stimulation, making careful calibration and monitoring essential for reliable data collection.

#### Tests for movement-evoked hypersensitivity

Less commonly, movement evoked hypersensitivity tests assess pain indirectly by evaluating animal’s response to challenging or potentially pain-evoking movements such as twisting, stretching or bending. These include, tail suspension, grip strength, rotarod, and lateral bending maze tests (Table 5).

The most common of these tests is grip strength assessments, which assesses animal sensitivity by stretching the spinal to identifying potential discomfort or weakness.^328^ Similarly, the tail suspension test evaluates spinal sensitivity by leveraging gravity to induce stretching of the spine.^313,329^ These assays are favored for its relative simplicity and minimal need for specialized equipment, however, they require technical consistency and careful handling to prevent stress-related artifacts, ensuring reliable and reproducible results.

In contrast, the rotarod tests assess balance and motor coordination by measuring the time an animal can remain on a rotating rod, reflecting pain-induced motor impairments.^273,293,330–332^ However, unlike other assays, rotarod testing carries high risk of animal injury, making it less ideal.

The lateral bending maze test evaluates flexibility and discomfort associated with movement-induced pain.^333^ Unlike other movement-evoked hypersensitivity assays, the lateral bending maze provides a more controlled environment, reducing external variables and allowing for more precise assessments of movement-related pain responses.

#### Tests for spontaneous pain-related behaviors

Finally, assays that monitor spontaneous behaviors – such as weight bearing, catwalk/gait analysis, and open field – offer valuable insight into pain-related alterations in natural behaviors and locomotion (Table 5).

Weight-bearing assays measure the distribution of weight on the limbs, allowing for the detection of asymmetries indicative of pain or discomfort.^333–336^ Slight shifts in the weight distribution can reflect compensatory behaviors following injury or intervention, thus indirectly assessing pain response. However, this test, however, requires highly sensitive equipment and some tester interpretation during analysis.

Catwalk and gait analysis systems record and analyze the animal’s walking pattern, assessing stride length, paw placement, and coordination changes resulting from pain.^285,333,337–339^ However, these tests are difficult for the animals to perform, as rodents do not typically walk in a straight line, but rather hop, or run, and insufficient step cycles will result in inaccurate data.

Open field tests provide insights into exploratory behavior, anxiety levels, and overall activity, which can be influenced by pain-induced alterations in mood or motivation.^333,340,341^ Unlike many of the other assays, the open field test provides a broad evaluation of both locomotor and behavioral activity levels and requires minimal training to perform. However, it is not ideal for detecting subtle changes which may occur in the early stages of LBP development and can easily be confounded by other variables such as anxiety, time of testing, and environmental conditions. Additionally, because results are indirect, data interpretation can be complex.

Biobehavioral testing provides valuable insights into pain mechanisms and responses in animal models, facilitating the development of effective pain management treatments. By utilizing a combination of these tests, researchers can comprehensively evaluate LBP development and uncover potential physiological and neurological changes associated with LBP.

## Current and future treatments

Current treatment guidelines for chronic LBP and degenerative disc disease involve symptom relief through intervention via medication and surgical therapy. Although these methods are useful for mitigating pain associated with IVDD, they are unable to reverse or halt the causes and progression of disc degeneration.^342^ Proposed clinical trials and hypotheses for future treatments look to address these shortcomings. Although there is optimism surrounding the potential of IVDD treatments for patient pain relief, further work needs to be done.

### Pain medications

A common practice for patients suffering from chronic LBP is the utilization of pain relief medication. Although effective at relieving pain in the short term, medications are not a long-term solution for patients suffering from chronic LBP as they do not address the root cause of pain and can lead to side effects from long-term use.^343,344^

#### Non-steroidal anti-inflammatory drugs

Non-steroidal anti-inflammatory drugs (NSAIDs) work by inhibiting cyclooxygenase (COX) enzymes, which normally function to convert arachidonic acid molecules into prostaglandins, causing swelling and an inflammatory response that leads to pain.^345–347^ Prostaglandins that are produced during a pro-inflammatory response increase blood flow to affected tissues. Further, prostaglandins, such as prostaglandin E_2_, cause pain by acting on peripheral sensory neurons and within the CNS.^348^ In the IVD, there is limited blood supply, which can limit the effectiveness of NSAID use. Despite this, NSAIDs have been found to be effective at providing short-term relief for patients with chronic LBP.^349^ NSAIDs are particularly desirable for patients because many forms are available over the counter, which makes it easier for patients to obtain them compared to other pain relief medications. As a whole, NSAIDs are widely tolerated by patients but do carry a risk of side effects, such as an increased risk of cardiovascular disease, gastrointestinal ulcers, and renal failure.^346,349,350^

#### Opioid medications

For patients experiencing significant levels of pain that cannot be alleviated through over-the-counter medication use, opioid pain medications are available via prescription. Most opioids work by eliciting analgesia through the mimicking of naturally produced opioid peptides in the body and binding to opioid receptors.^351,352^ Opioid receptors are GPCRs that are embedded in the cell membrane of cells found throughout the body.^353^ When stimulated, opioid receptors activate GPCR pathways, converting guanosine triphosphate (GTP) to guanosine diphosphate (GDP) and inhibiting adenylate cyclase, leading to increased levels of cyclic adenosine monophosphate (cAMP) intracellularly.^354^ Higher levels of cAMP lead to K^+^ channel hyperpolarization and the closure of Ca^2+^ channels, activating MAPKs.^354^ As a result of pathway activation, neurotransmitter release is reduced, decreasing the transmission of pain signals along both afferent and efferent pathways.^354^

Different opioid receptors exist, such as MOP (μ), DOP (δ), and KOP (κ) receptors, which each interact with different opioids that are naturally produced in the body and opioid medications.^354^ As a result, certain medications have higher affinities for specific receptors. Opioids act on opioid receptors by binding and stabilizing the active conformation of the receptor, leading to pathway activation and pain relief.^354^

Although these medications do provide patients with considerable pain relief, opioids have a substantial risk of addiction and chronic opioid use for patients.^355^ Furthermore, recent findings have found that patients who utilize opioids for symptom relief from chronic LBP are at an elevated risk of chronic opioid use compared to patients who use opioids for relief from other musculoskeletal diseases.^356^

#### Steroid injection

A more invasive medication available to patients is the use of epidural steroid injections. Corticosteroids are one of the more commonly used steroids, eliciting pain relief by mimicking cortisol.^342,357^ This prevents the release of arachidonic acid from cells and blocks the COX pathway, ultimately reducing inflammation.^342,357^ Current clinical trials are investigating whether the route of steroid administration for discogenic LBP can influence the quality of pain relief experienced by the patient. One such trial is investigating the efficacy of lumbar transforaminal epidural steroid injections.^358^ Despite this, there are many side effects that corticosteroids can cause, which may require further medications to be prescribed to reduce side effect severity.^359^ As a result, corticosteroids are recommended for short-term use and at low dosages.^359^ Furthermore, steroid injections for chronic LBP have not been shown to provide long-term relief for patients.^360^

#### Other medications in clinical trials

Other medications are also being investigated for future use in IVDD pain management. Pamidronate is a bisphosphonate that is classically used for treating hypercalcemia of malignancy and Paget disease of bone.^361^ Pamidronate works to inhibit bone resorption by osteoclasts.^361,362^ Bisphosphonates are a class of medications that mimic inorganic pyrophosphate and bind hydroxyapatite crystals.^361^ When osteoclasts utilize hydroxyapatite during bone resorption, pamidronate is left unbound, where it can act on farnesyl pyrophosphate synthase within the osteoclasts and induce apoptosis.^361^ Pamidronate has shown evidence of being effective at managing pain associated with bone diseases, and as such, researchers are looking to see if these benefits can be harnessed for use in discogenic LBP.^361,362^

Abaloparatide is a synthetic analog of parathyroid hormone-related protein (PTHrP). In an animal model of back pain in mice, researchers found that parathyroid hormone use effectively decreased disc degeneration.^363^ Abaloparatide has been used in the treatment of osteoporosis to increase bone mineral density and to slow and prevent disease progression.^364^

Alpha-2-Macroglobulin (A2M) is a naturally occurring macromolecule found in the extracellular matrix and acts as a protease inhibitor.^365^ One such protein that A2M targets and prevents the formation of is the cartilage degradation product, Fibronectin-Aggrecan complex (FAC).^366^ FAC has been identified as a potential cause of pain in patients with IVDD.^366^ As such, a clinical trial has been proposed to concentrate a patient’s A2M within their blood plasma and return the concentrate to the patient to target FAC.^366^ A2M prevents damage by FAC and other proteases by forming a tetrameric cage to encapsulate the protease and prevent further breakdown product formation.^365^

#### Surgery

Surgical intervention is considered for patients afflicted by severe symptoms of chronic LBP who do not experience relief from non-invasive treatment measures. Current surgical interventions include spinal fusion and total disc replacement.^342^ Despite the high rate of surgical treatment in chronic LBP patients, recent findings suggest that surgical intervention has little evidence of being beneficial for the patient.^367^

#### Spinal fusion

Spinal fusion is a popular surgical intervention for chronic LBP mediated by IVDD.^342^ Spinal fusion surgeries require the removal of the degenerated disc, cleaning of the IVD space, and implantation of a cage into the vacated IVD space to return the IVD space to its proper height.^342^ The spine is further stabilized with screws, plates, or rods to aid in the fusion process between adjacent vertebrae.^342^ There are many techniques regarding hardware placement into the vertebra that raise questions regarding the best technique for each patient’s unique circumstances.^368^ Questions include considerations for the type of hardware used, the insertion angles of the hardware, and the vertebral bony landmarks to which the hardware is anchored.^368^ Furthermore, this procedure can take place in either a minimally invasive or open surgery setting, each coming with its own costs and benefits.^342^ Minimally invasive surgical techniques are beneficial because of a decreased skin incision size, leading to less muscle and soft tissue injury during the surgical process and recovery.^342^ However, these procedures require specialized surgical equipment, and an increased time of operation compared to open surgery.^342^ Spinal fusion is considered successful when bone tissue is produced, connecting the vertebrae above and below the removed IVD. As a technique, spinal fusion is beneficial for pain relief in some patients while sacrificing spine mobility.^369^ Some studies have reported long-term consequences in patients who undergo spinal fusion, such as degeneration of the adjacent IVD.^370^ Furthermore, additional studies have reported few long-term benefits for patients who underwent the procedure and pointed to evidence of some patients requiring the use of opioids and other medications post-operatively for continued pain management.^371^

#### Total disc replacement

Total disc replacement is a surgical intervention for IVDD in patients with chronic LBP with the goal of relieving patient pain while retaining range of motion in the spine.^372,373^ In a total disc replacement, the entire IVD is removed and replaced with an artificial disc composed of metal endplates that fuse to the patient’s adjacent vertebral bodies and a plastic spacer to maintain height between the vertebrae.^373^ Clinical trials are being conducted to investigate the development of new artificial discs comparing their effectiveness to that of current models and spinal fusion techniques.^374–376^ In contrast to spinal fusion, total disc replacement enables the patient to retain a higher range of motion.^373^ Also, when directly comparing spinal fusion to total disc replacement, statistically significant findings were observed for short-term pain relief, disability, and quality of life, however, it was not considered clinically significant for the patient.^373^

### Platelet rich plasma

PRP is currently being studied as a potential treatment for musculoskeletal disorders, such as chronic LBP caused by IVDD.^377,378^ PRP refers to a portion of the patient’s blood obtained during a peripheral blood draw and is rich in platelets and growth factors, like PDGF, TGF, VEGF, EGF, FGF, CTGF, IGF-1.^379,380^ Previous research into IVDD has demonstrated that the use of growth factors commonly found in patients’ blood has yielded positive results in vitro and in vivo when studying effects on extracellular matrix synthesis and IVD cell proliferation.^380^ It has been demonstrated that PRP can provide similar benefits in a cell culture setting and in animal models, showing evidence of IVD height restoration and extracellular matrix production.^380^ Despite this, evidence surrounding the use of PRP in humans is highly debated. There is still much to understand about PRP and its effects, including limitations on a consensus PRP preparation method and the best route of PRP administration in a clinical setting.^379^

### Stem cell therapies

The utilization of stem cells for regenerative therapy in IVDD has shown encouraging evidence that they can be implemented in future treatments.^381–383^ Many hypotheses about stem cells and IVDD revolve around the transplantation of stem cells into the IVD and allowing for cell growth and proliferation to rebuild the IVD structure and replace the dead and dying cells. There is significant debate on the ideal stem cell type as well as methods to control the proliferation and growth of these cells in vivo. Current stem cell clinical trials are being conducted in humans using bone marrow-mesenchymal stem cells (BM-MSCs), umbilical cord-derived mesenchymal stem cells, and allogeneic mesenchymal stem cells.^384–387^ Research is also being conducted on the efficacy of NPCs and induced pluripotent stem cell-derived notochordal cells to determine if these cell populations may be better suited for treating discogenic LBP. Despite optimism regarding the potential of stem cell treatments, clinical trials have demonstrated that cells struggle to survive in the environment of the degenerated disc.^381–383^ As a result, further research needs to be done to better understand how stem cells can be utilized to treat IVDD and chronic LBP.

## Other minimally invasive therapies

Multiple clinical trials are underway looking into the use of injectable therapies to address discogenic LBP. Injectable therapies are hypothesized to be superior to surgical procedures due to their being less invasive in nature. Furthermore, the volume of tissue removal required and potential irreversible anatomical changes that result from surgical procedures like spinal fusions result in surgeries being viewed a last resort option. Injectables, such as morselized NP tissue allografts from cadaveric donors and cell-free hydrogel-based microgels aim to introduce therapeutic molecules and cells into the affected disc to relieve pain and halt the degeneration process.^388,389^ While these approaches show promise, injectable therapies remain in the early stages of development, with much still to be tested in regard to efficacy and long term durability. In addition, researchers are investigating combination strategies, utilizing the regenerative potential of cell therapies with the structural and bioactive support of biomaterials.

## Conclusion

LBP and IVDD are and will continue to be among the most prevalent musculoskeletal conditions in the world. Though a significant amount of research has been performed and had advanced our understanding of on IVDD and LBP, we have only scratched the surface of what mechanisms genetically, lifestyle-wise, molecularly, etc., are causing LBP to occur. However, recent advancements in diagnostic imaging, biological, and molecular techniques offer great promise for deepening our understanding and developing innovative therapeutic approaches.
